# Paternal Effect of the Nuclear Formin-like Protein MISFIT on *Plasmodium* Development in the Mosquito Vector

**DOI:** 10.1371/journal.ppat.1000539

**Published:** 2009-08-07

**Authors:** Ellen S. C. Bushell, Andrea Ecker, Timm Schlegelmilch, David Goulding, Gordon Dougan, Robert E. Sinden, George K. Christophides, Fotis C. Kafatos, Dina Vlachou

**Affiliations:** 1 Department of Life Sciences, Imperial College London, London, United Kingdom; 2 The Wellcome Trust Sanger Institute, Wellcome Trust Genome Campus, Hinxton, United Kingdom; National Institutes of Health, United States of America

## Abstract

Malaria parasites must undergo sexual and sporogonic development in mosquitoes before they can infect their vertebrate hosts. We report the discovery and characterization of MISFIT, the first protein with paternal effect on the development of the rodent malaria parasite *Plasmodium berghei* in *Anopheles* mosquitoes. MISFIT is expressed in male gametocytes and localizes to the nuclei of male gametocytes, zygotes and ookinetes. Gene disruption results in mutant ookinetes with reduced genome content, microneme defects and altered transcriptional profiles of putative cell cycle regulators, which yet successfully invade the mosquito midgut. However, developmental arrest ensues during the ookinete transformation to oocysts leading to malaria transmission blockade. Genetic crosses between *misfit* mutant parasites and parasites that are either male or female gamete deficient reveal a strict requirement for a male *misfit* allele. MISFIT belongs to the family of formin-like proteins, which are known regulators of the dynamic remodeling of actin and microtubule networks. Our data identify the ookinete-to-oocyst transition as a critical cell cycle checkpoint in *Plasmodium* development and lead us to hypothesize that MISFIT may be a regulator of cell cycle progression. This study offers a new perspective for understanding the male contribution to malaria parasite development in the mosquito vector.

## Author Summary

The unicellular protozoan parasites that cause malaria must undergo sexual development and subsequent proliferation in mosquitoes before they can infect humans and cause malaria. We characterized the first protein with paternal effect on the development of malaria parasites in the mosquito. This protein, which we named MISFIT, is produced in the progenitor cells of male gametes and found in the nuclei of these cells as well as in the nuclei of zygotes and their invasive forms, termed ookinetes. Disruption of the gene that encodes MISFIT leads to ookinetes with reduced DNA content, a defective secretory machinery and altered expression of various regulators of DNA replication and cell division. These mutant parasites stop developing immediately after traversing the mosquito gut, leading to malaria transmission blockage. Our study offers a new perspective for understanding the sexual development of malaria parasites in the mosquito vector, which leads to transmission of one of the most devastating diseases of mankind.

## Introduction

Malaria pathology is caused by asexual replication of apicomplexan parasites *Plasmodium* in the host bloodstream, but transmission between hosts requires sexual replication of parasites within mosquitoes. Subsets of sexually committed haploid merozoites escape each bloodstream replication cycle and differentiate into male and female gametocytes. Mature gametocytes are arrested in development until their uptake by a female mosquito during her blood meal. These cells reportedly have increased DNA content that may suggest selective gene amplification since genome replication does not occur during gametocytogenesis [1],[2],[3].

Gametogenesis begins within minutes of gametocyte ingestion into the mosquito gut. A male gametocyte undergoes three successive rounds of DNA replication producing eight haploid genome copies, initially confined within a persistent nucleus [2],[3]. Karyokinesis and cytokinesis, and consequent release of eight gametes are facilitated by cytoplasmic axonemes, each of which pulls one genome copy into the developing flagellate microgamete [4]. This process is known as exflagellation and regulated by calcium-dependent signaling. Two key regulators have been identified in the rodent malaria parasite *Plasmodium berghei*: the cyclin-dependent kinase CDPK4 controls the initiation of genome replication [5] while a downstream mitogen-activated protein kinase, map-2, regulates the onset of cytokinesis and release of microgametes [6],[7]. In parallel, activated female gametes (macrogametes) enlarge and emerge from the red blood cells.

Fertilization begins with gamete adhesion, followed by plasma membrane fusion and entry of the male nucleus and axoneme into the macrogamete [8]. The P48/45 protein on the surface of microgametes is essential for the initial fertilization stages [9]. In *P. berghei*, fusion is mediated by the male sterility factor HAP2 [10], also known as generative cell specific 1 (GCS1) [11]. Pronuclei fusion is followed by a meiotic replication cycle that, in the absence of nuclear division and cytokinesis, renders the zygote tetraploid [2],[8]. Initiation of meiotic DNA replication is regulated by female-specific expression of the NIMA (never-in-mitosis/Aspergillus)-related kinase, Nek-4 [6],[12].

Within the next 12–24 hours, the zygote elongates and develops into the mature ookinete. This process is associated with formation of the polar ring that acts as a microtubule-organizing centre (MTOC) at the ookinete apical pole, organizing a network of subpellicular microtubules [13]. This network regulates polarized trafficking of secretory organelles including micronemes and is linked to the ookinete actomyosin motor, together facilitating the motility and invasive ability of the ookinete that escapes the blood bolus and traverses the midgut epithelium.

Further DNA replication and chromosome segregation occur only after the ookinete reaches the basal lamina, where it rounds up and transforms into the sessile oocyst. Morphological changes associated with this transition include loss of the apical complex and subpellicular microtubules, and parasite encasement in a proteinaceous capsule [4]. As development progresses, the nucleus becomes polyploid through multiple rounds of endomitosis while the plasmalemma invaginates forming sporoblasts. In the mature oocyst, synchronous nuclear divisions direct sporozoite budding-off from the sporoblasts, generating thousands of haploid sporozoites. These are released into the mosquito haemocoel, invade the salivary glands and infect new hosts during subsequent mosquito blood meals.

Throughout *Plasmodium* development, DNA replication, chromosome segregation and cytokinesis are uncoupled. The molecular mechanisms underpinning and regulating this unorthodox cell cycle remain poorly understood. Here we report the discovery and characterization of MISFIT, a novel nuclear formin-like protein with critical functions in *P. berghei* sexual and sporogonic development in the mosquito. Formins are involved in the regulation of the actin and microtubule networks during mitosis, meiosis, cell polarization and vesicular trafficking [14]. The *misfit* gene is expressed in gametocytes, and the protein is detected in the nucleus of male gametocytes, zygotes and ookinetes. Disruption of the gene leads to ookinetes with incomplete genome content (approximately 3C-values) and defects in the transcription of putative cell cycle regulators. These ookinetes invade the mosquito midgut but are arrested in development during their subsequent transformation into oocysts, resulting in transmission failure. Our data suggest involvement of MISFIT in the regulation of microtubule remodeling during DNA replication and chromosome segregation in mitosis in male gametocytes and/or meiosis in zygotes. Furthermore, we establish the ookinete-to-oocyst transition as a cell cycle checkpoint of *Plasmodium* development. Mutant ookinetes also have severely reduced numbers of micronemes, but do invade the mosquito midgut. This points to an intriguing function of MISFIT in vesicular trafficking and challenges the long-held view that micronemes are indispensable for midgut invasion. Genetic crosses reveal an absolute requirement for a functional male allele of *misfit* in *Plasmodium* development in the mosquito. This is the first gene with paternal effect on *Plasmodium* post-fertilization development. Its discovery opens new research avenues for understanding the male contribution to parasite development in the mosquito and ultimately malaria transmission.

## Results

### *P. berghei* MISFIT is a novel nuclear formin-like protein

We searched published proteomic data for putative nuclear proteins expressed during *P. berghei* development in the mosquito to investigate the mechanisms that regulate the parasite sexual and early sporogonic development in the vector. Two separate studies detected the Pb000064.01.0 protein in mature oocysts [15] and in gametocytes [6], respectively. We named this protein MISFIT for reasons explained below. It is an 180 kDa protein bearing a Formin Homology 2 (FH2) domain, the defining feature of formins, and a nuclear localization signal (NLS) (Figure S1A). Using a domain prediction algorithm for proteins associated with nuclear functions [16], we detected a putative kinase domain at the N-terminus of MISFIT, downstream of the NLS. A basic amino acid region at the C-terminus of MISFIT shows similarities to the self-inhibitory Diaphanous Autoregulatory Domain (DAD) present at the C-termini of Diaphanous-related formins (DRFs) [17]. Two formins with orthologs in all plasmodia have been identified previously in *Plasmodium falciparum* [18],[19]. Unlike MISFIT, these two and many other formins encompass a proline-rich FH1 domain that interacts with profilin-bound actin monomers, thus accelerating actin filament elongation [14].

Bioinformatic searches revealed the presence of orthologous MISFIT proteins in diverse *Plasmodium* species. Their identity with *P. berghei* MISFIT ranges from 92% in *Plasmodium yoelli* to 32% in *P. falciparum* and *Plasmodium vivax*. Similarities are highest in the FH2 domain and a central region of ∼180 amino acids (Figure S2). Putative N-terminal kinase-like domains are also predicted for *P. yoelli* and *P. falciparum* MISFITs. Phylogenetic analysis of the FH2 domains in all the apicomplexan formin-like proteins revealed highest similarity between *Plasmodium* MISFITs and *Cryptosporidium parvum* Formin 4 (Figure S1B).

### *In vivo misfit* gene expression in infected mosquitoes

**Figure 1:**
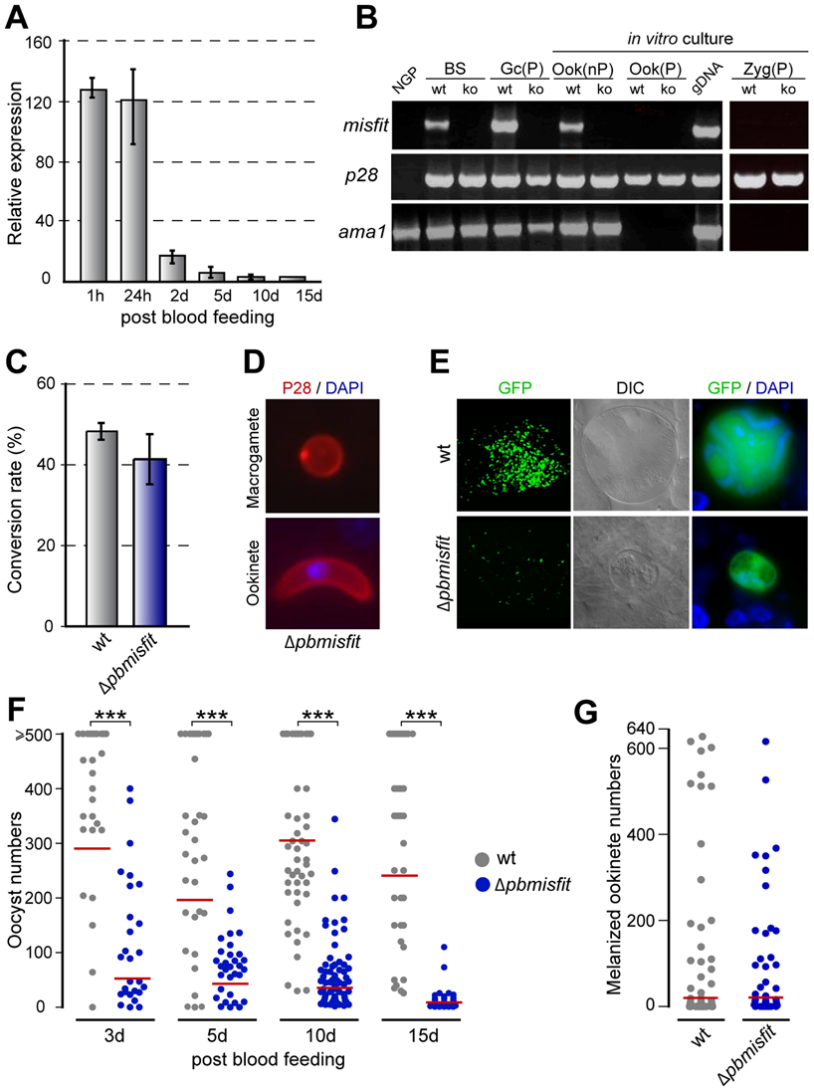
*Misfit* gene expression and phenotypic analysis of *Δpbmisfit* mutants. (A) Relative abundance of *misfit* transcripts in *P. berghei*-infected *A. gambiae* midguts, assayed by quantitative real-time RT-PCR. The constitutively expressed *gfp* transgene was used as reference. The average expression and standard errors of three independent biological replicates (different batches of mosquitoes fed on different blood sources) are shown. The results of each biological replicate are the average of two technical replicates. (B) RT-PCR analysis of *misfit* in non-purified (nP) and purified (P) *wt* and *misfit* ko parasite populations. Genes encoding the female/zygote sexual stage protein, P28, and the blood-stage protein, Ama1 (apical membrane antigen 1), served as stage-specific and loading controls. NGP, non-gametocyte producing strain; BS, mixed asexual and sexual blood stages; Gc, gametocytes; Ook, ookinetes; gDNA, genomic DNA; Zyg, zygote. (C) Macrogamete to ookinete conversion assay in control *wt* and *Δpbmisfit* mutant parasites. (D) *In vitro* cultured *Δpbmisfit* parasites stained for P28 (red) and DNA (DAPI, blue). (E) Microscopy images of 15-day-old *wt* and *Δpbmisfit* oocysts in *A. gambiae* midguts (first column ×10 objective; second and third columns ×63 objective). GFP-expressing parasites appear green. DIC, differential interference contrast. (F) Distribution of *wt* and *Δpbmisfit* oocyst numbers in midguts of *A. stephensi* mosquitoes, at day 3, 5, 10 and 15 post blood feeding. The geometric means of oocyst numbers (red line) are shown. Highly significant reduction (1-way ANOVA, P<0.001, ***) of *Δpbmisfit* oocyst numbers compared to *wt* controls is detected at all time points. Oocyst numbers equal or higher than 500 were individually enumerated and used to calculate the means. The full analysis is presented in Table S2. (G) Ookinete invasion assay in *CTL4* kd *A. gambiae*. Distribution and geometric means of melanized ookinete intensities are shown. No statistical difference is detected between *wt* and *Δpbmisfit* parasites.

We used quantitative real-time RT-PCR to investigate *in vivo* the stage-specific transcription of the *misfit* gene in midguts of *A. gambiae* mosquitoes fed on *P. berghei*-infected mice. Abundant transcripts were detected 1 h and 24 h post blood feeding (pbf), corresponding to the beginning and end of parasite development in the midgut lumen, respectively (Figure 1A). After invasion across the midgut epithelium and oocyst formation on the basal midgut wall, *misfit* expression drops significantly and is barely detectable after day-5. RT-PCR analysis revealed *misfit* transcripts to be present in mixed asexual and sexual blood-stage parasites and purified gametocytes, but not in purified zygotes or ookinetes (Figure 1B). Thus, transcripts observed in mosquito midguts 24 h pbf probably derive not from zygotes or ookinetes, but from earlier developmental stages that persist in the blood bolus. Data presented later in the manuscript revealed that expression of *misfit* takes place in gametocytes.

### Disruption of *misfit* blocks ookinete-to-oocyst transition

Mutant *P. berghei* were generated by replacing part of *misfit* with a modified *Toxoplasma gondii* pyrimethamine resistance cassette in the Pbc507 GFP-expressing parasite reference line [20]. Integration of this disruption cassette was verified by pulse field gel electrophoresis, and generation of clonal *Δpbmisfit* parasites was confirmed by PCR and Southern blot analysis (Figure S3). Compared to wild-type (*wt*) controls, the *Δpbmisfit* knockout (ko) mutant parasites exhibited normal development of asexual blood stages, mature gametocytes and male gametes. The conversion rate of mutant macrogametes to ookinetes was also comparable to the controls (Figure 1C), and both stages displayed normal morphology and surface distribution of the P28 protein [21] (Figure 1D). However, sporogonic development of *misfit* ko parasites in both *A. gambiae* and *A. stephensi* mosquitoes was severely compromised: mature oocysts were extremely rare and small in size (Figure 1E and Table S1). Furthermore, unlike the highly organized nuclei of *wt* oocysts, the few *Δpbmisfit* oocysts that persisted to day-15 pbf showed much reduced and diffuse DNA staining (Figure 1E), indicating possible defects in DNA replication and/or chromosome segregation.

Oocyst numbers were severely reduced already at day 3 pbf (Figure 1F and Table S2), indicating that MISFIT is essential for the ookinete-to-oocyst developmental transition. This reduction became progressively more obvious, as mosquitoes gradually cleared defective oocysts. Direct membrane feeding of mosquitoes with a suspension of *in vitro* produced ookinetes in uninfected blood yielded similar results (data not shown). These data were independently corroborated by disruption of *misfit* in the *P. berghei* 2.34 ANKA genetic background (data not shown).

We used *A. gambiae* C-type lectin 4 (*CTL4*) knockdown (kd) mosquitoes to investigate whether mutant *Δpbmisfit* ookinetes can invade the midgut epithelium. CTL4 is an inhibitor of melanization, and its depletion by RNAi causes mosquitoes to melanize ookinetes soon after they reach the basal sub-epithelial space, where they encounter hemolymph components that are essential for melanization [22]. The numbers of melanized *Δpbmisfit* and *wt* control ookinetes were comparable, indicating that the mutant parasites are not invasion deficient (Figure 1G). Furthermore, *Δpbmisfit* ookinetes injected directly into the mosquito hemocoel failed to rescue the ko phenotype (Table S3). Taken together our data clearly indicate that the *Δpbmisfit* phenotype is determined at the onset of oocyst development, and not by failure of midgut invasion.

### MISFIT localizes to the nuclei of male gametocytes, zygotes and ookinetes

**Figure 2:**
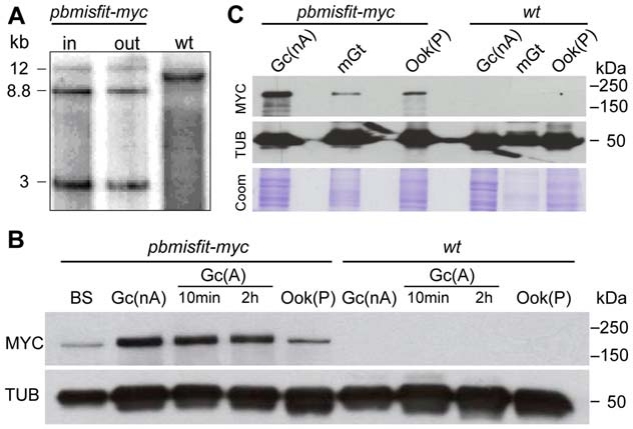
Expression of MYC tagged MISFIT in transgenic *P. berghei*. (A) Southern blot analysis of input and output (back-bite) *pbmisfit-myc* parasite populations demonstrates population purity and stability of the tagged locus that was generated as shown in Figure S3. Genomic DNA was digested with EcoRI and a 3′ UTR fragment of *misfit* was used as probe. Insertion of the transgenic cassette resulted in a 3 kb digestion product, which is absent from *wt* parasites. The 8.8 kb band represents a tandem insertion of two tagging vectors in the target *misfit* locus. The *wt* band detected for the native *misfit* locus is absent from *pbmisfit-myc* populations. (B) Western blot analysis of transgenic *pbmisfit-myc* parasite populations using an anti-MYC antibody. *Wt* parasites were used as a control. Tubulin (TUB) detected with a mouse monoclonal antibody against *Trypanosoma brucei* alpha-tubulin (tat1) was used as a positive control. BS, mixed asexual and sexual blood stages; Gc(nA), non activated gametocytes; Gc(A), activated gametocytes 10 min or 2 h post-activation; Ook(P), purified ookinetes. (C) Western blot analysis of *pbmisfit-myc* microgametes (mGt) using an anti-MYC antibody. Gc(nA) and Ook(P) extracts were used as a control. TUB was used as internal control, and Coomassie (Coom) stained extracts as a loading control. Numbers indicate protein size scale in kDa.

We used a single homologous recombination approach to generate a transgenic *P. berghei* line (*pbmisfit*-*myc*) expressing a C-terminal MYC-tagged MISFIT protein (Figure S4) and investigate the pattern of MISFIT protein expression. Mosquitoes infected with tagged parasites exhibited intensities of sporulating oocysts comparable to those in control infections (data not shown), indicating that the tagged protein is fully functional. Southern blot analysis of the parasite input (blood-stage parasites from gametocyte-donor mice) and output (blood-stage parasites from sporozoite-recipient mice) verified that developmental normality is not due to *wt* contaminants (Figure 2A). Western blot analysis using an anti-MYC antibody revealed high MISFIT levels in gametocytes, both before (non-activated) and after (activated) induction of gametogenesis, and lower levels in purified ookinetes (Figure 2B). In conjunction with the absence of *misfit* transcripts from zygotes and ookinetes (Figure 1B), these data suggest protein carry over from earlier developmental stages. Indeed, the protein was also detected in purified male gametes (Figure 2C).

**Figure 3:**
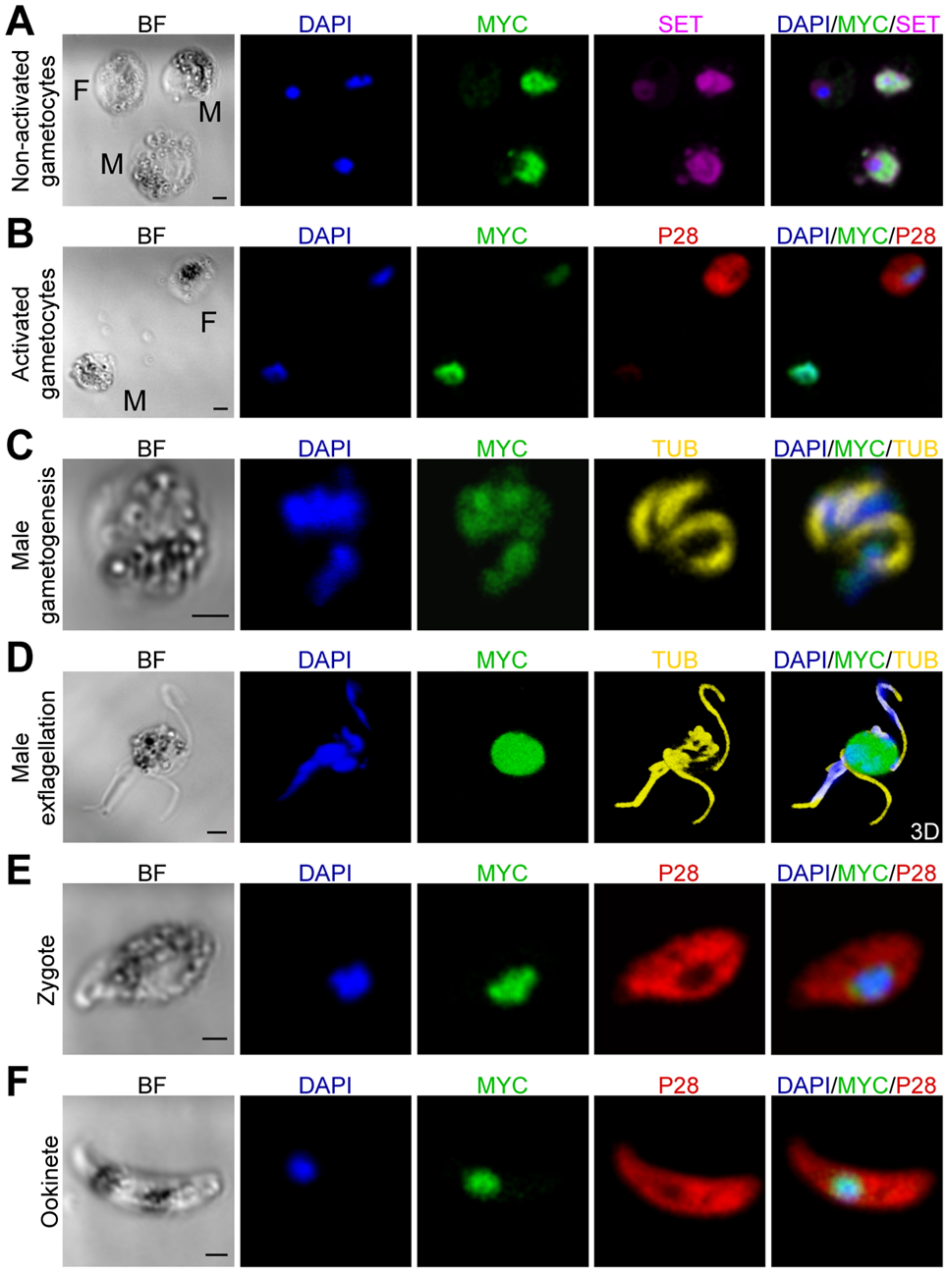
MISFIT protein expression, localization and distribution. (A–F) Immunofluoresence images from confocal sections of fixed *pbmisfit-myc* parasite stages. Images show DAPI staining of DNA in blue and the following antibody stainings: anti-MYC of MISFIT-MYC protein in green, anti-SET in violet, anti-P28 in red, and anti-Tubulin (TUB) in yellow. Fluorescent images of the exflagellating male gametocyte (D) are 3D reconstruction of a confocal stack after deconvolution. Scale bars in bright field (BF) images correspond to 2 µm. M, Male; F, Female.

Immunofluorescence assays in *pbmisfit*-*myc* parasites provided clear insights into the sub-cellular localization and putative function of MISFIT. Consistent with its NLS prediction, MISFIT is localized in the nuclei of male gametocytes (activated and not), zygotes and ookinetes (Figure 3). The presence of MISFIT in the male gametocyte nucleus was corroborated by co-localization experiments with SET (Figure 3A), a protein putatively involved in chromatin dynamics, which also strongly accumulates in male gametocytes [23]. Importantly, MISFIT localization did not perfectly match the DNA staining; it exhibited broader, sometimes polarized distribution, indicating that the protein is not a ubiquitous component of chromosomes. A weak, slightly above background signal in female gametocytes (Figure 3B), in conjunction with earlier proteomic data [6], suggests the possibility of low protein expression in females. MISFIT was not detected in asexual blood stage trophozoites or schizonts (data not shown).

In male gametogenesis, each round of DNA replication is followed by segregation of the haploid genomes to different poles of the compartmentalized nucleus [8]. During this process, MISFIT-MYC staining appears to follow the distribution of DNA (Figure 3C), possibly suggesting MISFIT involvement in chromosome segregation, which would be consistent with the putative function of formin-like proteins in regulating the microtubule cytoskeleton. Contrary to the clear detection of MISFIT-MYC in male gametes by western blot (Figure 2C), immunofluorescence staining could not confirm the presence of this protein in emerging (Figure 3D) or released male gametes (data not shown). In all confocal observations of exflagellating male gametocytes, MISFIT-MYC staining was confined within the parental gametocyte nucleus. The nuclear staining of MISFIT in zygotes and ookinetes resembled that in male gametocytes, with a broader pattern than that of the DNA staining and sometimes polarized or peripheral distribution (Figure 3E, 3F).

### Strict requirement for functional male *misfit* allele

**Figure 4:**
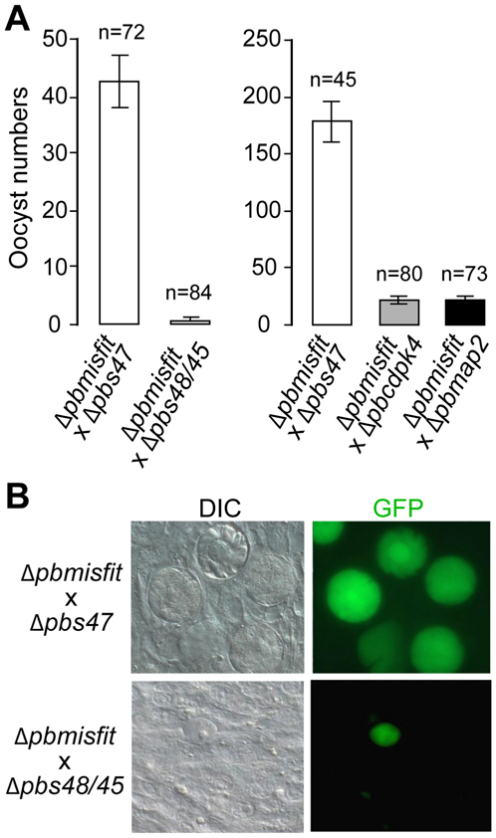
Genetic complementation experiments in *misfit* ko parasites. (A) Genetic crosses of *Δpbmisfit* with a female gamete deficient (*Δpbs47*) or male gamete deficient (*Δpbs48/45*, *Δpcdk4* and *Δmap2*) *P. berghei* mutants. Bars show mean 12-day-old oocyst numbers in midguts of *A. stephensi* mosquitoes. Left panel shows infections performed via membrane-feeding on ookinetes in *in vitro* cultures that were initiated with blood of mice co-infected with the indicated parasite mutants. The right panel shows infections of mosquitoes that directly fed on co-infected mice. Standard errors are shown; n indicates the number of midguts. (B) Microscopy observations of hybrid *Δpbmisfit*/*Δpbs47* and *Δpbmisfit*/*Δpbs48/45* oocysts (green) developing on *A. gambiae* midguts 12 days pbf. All images were obtained at 63× magnification. DIC, differential interference contrast.

Since the effect of MISFIT on parasite development is manifested after fertilization, at the ookinete-to-oocyst transition, we investigated whether inheritance of female or male *wt misfit* alleles by the zygote would be sufficient to rescue the ko phenotype. We performed genetic crosses between *Δpbmisfit* parasites and *P. berghei* mutants deficient either in female (*Δpbs47*) or in male (*Δpbs48/45*) gametes [6],[9],[24]. The results revealed a strict requirement for a functional male copy of *misfit* (Figure 4 and Table S4). Crosses between *Δpbmisfit* and *Δpbs47* mutants yielded oocysts of normal number, size, morphology, and capability to sporulate. In contrast, crosses between *Δpbmisfit* and *Δpbs48/45* parasites invariably produced oocysts exhibiting the *misfit* ko phenotype. The requirement for a male *misfit* allele was corroborated by *Δpbmisfit* crosses with additional female or male gamete deficient mutants carrying functional *misfit* genes: *Δpbnek4* [6], *Δpbmap2* [7] and *Δpbcdpk4* [5]. Control crosses of *Δpplp5* mutants [25] with the gamete deficient strain *Δcdpk4* confirmed that the paternal effect is specific to *Δpbmisfit* parasites (Table S4). *Pplp5* is required post-fertilization, during midgut invasion and its ko phenotype can thus be rescued by both maternal and paternal *wt pplp5* alleles [25],[26]. Based on these data, we named the gene *misfit* for male-inherited sporulation factor important for transmission.

### *Misfit* ko ookinetes have less DNA than *wt*

**Figure 5:**
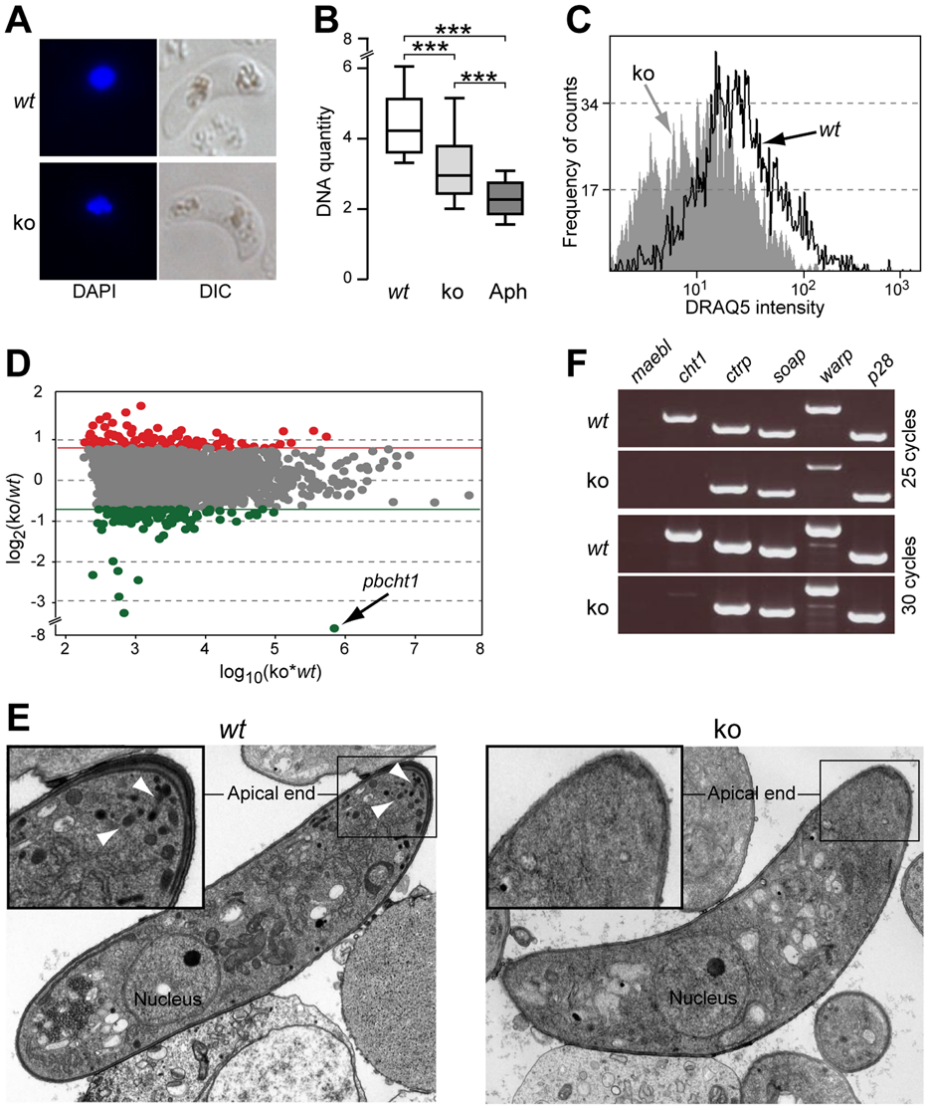
*Misfit* ko ookinetes show less DNA, altered transcriptome and defective micronemes compared to *wt*. (A) Microscopy images of *wt* and *misfit* ko ookinetes stained with DAPI. (B) Box plots showing DNA quantity levels of *wt*, *misfit* ko and aphidicolin-treated (Aph) ookinetes. DNA quantity is determined by the ratio of ookinete to asexual blood stage (haploid) measurements of DAPI-staining intensities. Boxes are divided by the arithmetic mean to upper and lower quartiles, respectively. Upper and lower whiskers indicate distribution ranges. Results from ANOVA t-test comparisons are shown (***, P<0.001). (C) Analysis of fluorescence intensities by flow cytometry of *wt* and *misfit* ko ookinetes stained with the DNA-intercalating dye DRAQ5. (D) Microarray Ratio-Intensity (R-I) plot showing differential gene expression measurements between control *wt* and *misfit* ko ookinetes. Measured intensities are plotted as a function of the logarithm of their product. Red and green dots represent genes that are up or downregulated by at least 1.7 fold, respectively, in *misfit* ko compared to *wt* ookinetes. Grey dots represent genes that do not show significant regulation. (E) Transmission electron micrographs (TEM) of *wt* (left panel) and *misfit* ko (right panel) ookinetes. Insets show magnifications of the ookinete apical ends, depicting the severe microneme reduction in *misfit* ko compared to *wt*. Normal *wt* micronemes are indicated with white arrowheads. (F) RT-PCR of genes encoding known micronemal proteins. PCR reactions were performed at 25 and 30 cycles, respectively, to highlight expression differences. *Maebl* encodes a micronemal protein of sporozoites and merozoites, but not of ookinetes, and was used as a negative control. The ookinete-expressed *p28* does not encode a micronemal protein and was also used as a control.

Microscopy observations suggested that *Δpbmisfit* ookinetes have reduced amounts of DNA (Figure 5A). We treated parasites 1 h post fertilization with the DNA polymerase inhibitor aphidicolin to inhibit meiotic DNA replication in zygotes and produce ookinetes with diploid instead of tetraploid genomes [2]. We then used DAPI-staining measurements of microscopy images (Figure 5B) to compare the amounts of DNA between *Δpbmisfit*, aphidicolin-treated and untreated ookinetes. These measurements were normalized to the DNA content of asexual haploid parasites. Importantly, *Δpbmisfit* ookinetes exhibited an intermediate C-value of 3 indicating that in the absence of MISFIT, meiotic DNA replication was initiated but either was aborted prior to completion, or the starting DNA material was less than in the *wt* controls. Flow cytometry measurements of *Δpbmisfit* and *wt* ookinetes following treatment with a fluorescent DNA-intercalating dye DRAQ5 confirmed the mutant is DNA deficient (Figure 5C).

Infections of control and *CTL4* kd *A. gambiae* with aphidicolin-treated ookinetes revealed that these ookinetes invade the mosquito midgut but fail to transform into oocysts, exhibiting a phenotype indistinguishable from that of *misfit* ko parasites (Figure S5). These results indicate that meiotic DNA replication in zygotes does not affect ookinete development and midgut invasion, but is critical for transforming ookinetes into oocysts, perhaps at initiation of mitosis in the oocyst.

### Microarray analysis of *misfit* ko ookinetes

We investigated whether absence of MISFIT and the consequent effects on ookinete DNA content and meiotic replication also affect the ookinete transcriptome. Hybridizations of long oligonucleotide microarrays representing 5,361 genes encoded by the *P. berghei* genome identified 231 genes as at least 1.7-fold up- or downregulated in the *in vitro* cultured *misfit*-deficient ookinetes, as compared to *wt* controls (Figure 5D and Table S5). INTERPRO domain scans indicated that several such genes might function in cell cycle regulation and DNA replication. They include the transcription factor Myb1 that reportedly regulates the *P. falciparum* intra-erythrocytic cell cycle [27], a member of the regulator of chromosome condensation superfamily [28], DNA and RNA helicases, nucleoside biosynthesis enzymes, DNA repair enzymes, a cyclin, and eight kinases, some predicted to be cyclin-dependent. Genes involved in the regulation of transcription and translation were also included, e.g. *hmgb2*, a known important regulator of sexual gene expression in *P. yoelii* [29].

However, the most affected gene by far (160-fold downregulated) was the chitinase-encoding *pbcht1* gene. Chitinase is a micronemal protein implicated in mosquito midgut invasion [30]. Orthologous *P. falciparum* and *Plasmodium gallinaceum* enzymes are thought to facilitate penetration of the chitinaceous peritrophic matrix that envelops the blood bolus in the midgut [31].

### *Misfit* ko ookinetes are deficient in micronemes

We used electron microscopy to investigate whether disruption of *misfit* causes morphological changes to ookinetes, undetectable by light microscopy. The results revealed that *misfit* ko ookinetes exhibit a severe defect in micronemes (Figure 5E), the specialized organelles in the ookinete apical complex that secrete soluble or cell surface molecules, including CHT1 [31]. Of 38 analyzed sectioned profiles of mutant ookinetes, only one displayed normal micronemal content; 21 had greatly reduced microneme numbers, and 16 had none at all.

We used RT-PCR to investigate whether the defect in microneme formation is accompanied by downregulation of additional micronemal protein encoding genes (Figure 5F). Of four examined genes, *warp* [32] was also downregulated in the absence of MISFIT, but much less so than *cht1*. The expression of *ctrp* [33],[34] and *soap* [35] was unaffected.

## Discussion

Apicomplexan parasites require sexual reproduction to complete their complex life cycles. Sexual reproduction and subsequent sporogonic development of *Plasmodium* in mosquitoes ultimately result in malaria transmission. Understanding the genetic and molecular basis of transmission could lead to novel approaches for tackling one of the most devastating diseases of mankind.

To date, three distinct classes of genes have been identified with critical functions in *Plasmodium* sexual and early sporogonic development. The first class includes genes such as *P48/45* [9] that are expressed in gametocytes and playing essential roles in gamete development or fertilization. The second class encompasses genes expressed *de novo* in the zygote, e.g. *ctrp* and *cht1*; they are typically implicated in ookinete motility and invasion. The third class comprises two subclasses of genes showing maternal effects. One subclass includes genes such as *P25* and *P28*, which produce transcripts that are translationally repressed by the DOZI complex and only released for translation in the zygote [36]. The second subclass includes genes transcribed and translated in the female gametocyte but showing mutant phenotypes only post-fertilization, e.g. the *LAP* genes (also known as *PCCp*) with phenotypes manifested during oocyst development [24],[37]. The molecular basis of these maternal effects is not well understood.

The characterization of *misfit* hereby establishes a fourth class of genes that are critical for sexual and early sporogonic development. *Misfit* is the first gene with paternal effect on *Plasmodium* post-fertilization development. Disruption of the gene results in ookinetes that invade the mosquito midgut but are arrested in development during their transformation to oocysts, thus blocking transmission. Genetic crosses revealed that the functional male allele of *misfit* alone is necessary and sufficient for normal parasite development and subsequent transmission to the host.

*Misfit* transcripts are restricted to gametocytes, but the protein is more broadly distributed: it is found not only in the male gametocyte but also in the zygote and the ookinete. Thus the paternal effect of *misfit* on the post-fertilization stages can be due to a knock-on effect caused by the protein function in the male gametocyte, paternal inheritance of the protein to the zygote, or both. Indeed MISFIT is detected in the male gamete, and therefore paternal inheritance is possible. However, strong MISFIT staining in the zygote and ookinete leaves open the possibility of additional *de novo* protein synthesis after fertilization. Such expression would be temporally limited, as transcripts are not detected in 8-hour zygotes. To explain the paternal effect, such *de novo* expression would either be insufficient for rescuing the mutant phenotype or would occur only in the male allele, due to genetic imprinting. Epigenetic mechanisms of transcriptional regulation have been described for the *P. falciparum var* genes [38] and are thought to be important throughout the Apicomplexa [39]. Overall, the discovery of *misfit* opens unprecedented opportunities to study the male gamete contribution to *Plasmodium* development.

MISFIT contains an FH2 domain, the defining feature of formins, a family of proteins that regulate the dynamic remodeling of the cytoskeleton in eukaryotic cells [14],[40]. The diverse functions of formins include regulation of actin nucleation and polymerization, orientation of the MTOC and spindle alignment at mitosis and meiosis, stabilization of microtubules, cell polarity and vesicular trafficking [41],[42],[43],[44]. Two formins have been previously identified in the human malaria parasite, *P. falciparum*, and their orthologs exist in all plasmodia [18],[19]. Both proteins, like many of the known formins, contain an FH1 domain that interacts with profilin to bring actin monomers to the polymerizing filament. Indeed, PfFormin1 regulates actin polymerization and localizes at the parasite-erythrocyte moving junction during invasion [18]. Apart from the FH2 domain, MISFIT does not share additional domains with the other two *Plasmodium* formins and thus is unlikely to share a similar function. Furthermore, MISFIT has an NLS, a DAD-like motif found in DRFs [17] and an unclassified kinase-like domain that is predicted to exist in nuclear proteins [16]. Recently, two formins with kinase C1-like domains were identified in the amoeba [45]; they localize with the spindle during mitosis and regulate DNA content and cell division.

Loss of MISFIT function results in ookinetes with reduced DNA content. This finding, in conjunction with MISFIT expression in the male gametocyte, its nuclear localization and distribution, and its domain composition suggest a putative role of this novel formin-like protein in regulating the mitotic spindle during *Plasmodium* male gametogenesis. During mitotic DNA replication in the male gametocyte, the absence of MISFIT may affect the overall organization of the spindle or destabilize its microtubules, resulting in gametes carrying incomplete haploid genomes. As DNA synthesis and gametogenesis occur within minutes, mitotic checkpoints are unlikely to exist [46]. Indeed, it has been observed that a significant subset of *wt* male gametes lack nuclei [47]. Carrying less DNA may not compromise exflagellation and the fertilization capability of male gametes, but could affect meiotic chromosome segregation in the zygote, leading to checkpoint implementation and developmental arrest at the initiation of endomitosis in the oocyst. In support of this hypothesis, aphidicolin-treated zygotes that do not undergo meiotic DNA replication exhibit a phenotype similar to that of *misfit* mutant parasites: they form ookinetes that invade the mosquito midgut successfully but are developmentally arrested at the onset of oocyst transformation. As MISFIT is also found in the nucleus of zygotes and ookinetes, an additional role of this protein in meiotic DNA replication and spindle microtubule remodeling is also possible.

In contrast to higher eukaryotes, the nuclear envelope of *Plasmodium* is maintained throughout the nuclear divisions (endomitosis), and spindles do not originate from typical cytoplasmic centrioles. Instead, intranuclear spindles are organized by centriolar plaques located at the inner side of the nuclear membrane and originate from an amorphous cytoplasmic MTOC that transforms during mitosis into a structured kinetosome embedded in the nuclear envelope [47]. These atypical features of the *Plasmodium* cell cycle are in accordance with the nuclear localization of MISFIT, as opposed to the typical cytoplasmic localization of formins. One exception is an isoform (mDia2) of a mammalian Diaphanous formin that was recently shown to also contain an NLS and shuttle between the cytoplasm and the nucleus [48]; the significance of this behavior of mDia2 remains unknown.

The basic molecular mechanisms regulating the atypical *Plasmodium* cell cycle remain unclear, and developmental checkpoints similar to those described in higher eukaryotes have not been identified [46]. A lack of specificity in the cyclin/CDPK pairing is believed to relate to a less conserved and more flexible role of apicomplexan cyclins compared to higher eukaryotic cells [49]. Regardless of the exact function of MISFIT in mitosis and/or meiosis, our data suggest that the ookinete-to-oocyst transition serves as a checkpoint of *Plasmodium* cell cycle progression in the mosquito. Parasites that fail to successfully overcome this checkpoint are developmentally arrested and progressively cleared by the mosquito. Thus, the characterization of MISFIT provides a new perspective for studying the *Plasmodium* cell cycle regulation and its checkpoints. Our microarray data showed that genes encoding a cyclin, several CDPKs and additional putative cell cycle regulators are differentially regulated in *misfit* mutant ookinetes and are key candidates for being involved in these processes.

The finding that *misfit* ko ookinetes have severely reduced micronemes is intriguing. Micronemes are the only known specialized secretory organelles of the ookinete and their secretions are thought to be important for host-cell recognition, binding and motility during parasite invasion [50],[51]. Hence, the ability of *misfit* mutant ookinetes to invade the mosquito midgut challenges the long-held view that micronemes are essential for mosquito midgut invasion.

Micronemes are synthesized *de novo* in the Golgi and translocate apically by using filamentous connections with sub-pellicular microtubules [3],[51]. An obvious hypothesis would be that the microneme defect relates to the putative function of MISFIT in microtubule remodeling. However, sub-pellicular microtubules are organized in the cytoplasm by a circular MTOC known as the apical polar ring, whereas MISFIT is found only in the nucleus. The structure of the apical polar ring resembles the microgamete MTOC that organizes the formation of the mitotic spindle and axoneme [47]. It has not been established to date whether the apical polar ring is of maternal or paternal origin; it would be tempting to link the microneme phenotype of *misfit* with inheritance of a defective MTOC by the male gamete, but remains a subject for future research.

The microneme deficit phenotype may also be due to changes in the expression of genes directly implicated in microneme formation. Indeed, our microarray experiments identified genes with putative functions in vesicle biogenesis and trafficking as being differentially regulated in *misfit* mutant ookinetes. Defective micronemes could in turn result in protein accumulation in the Golgi and generate negative signals that would downregulate the transcription of genes encoding micronemal proteins such as CHT1 and WARP. However, such feedback regulatory mechanisms are as yet unknown in *Plasmodium*. An alternative hypothesis is that the micronemal phenotype is caused by reduced production of cargo, e.g. CHT1. This would be consistent with the finding that depletion of Pfg377 from *P. falciparum* female gametocytes leads to great reduction of secretory osmiophilic bodies [52].

## Materials and Methods

### Parasite cultivation and purification and mosquito infections

*P. berghei* strains ANKA 2.34 and 2.33 (non-gametocyte producer), and the GFP-expressing reference lines 29c12 [53] and 507 [20] were propagated in mice using standard protocols. Parasite handling and purification of asexual and sexual blood stage parasites, male microgametes and ookinetes were performed as described [5],[10],[33],[54]. For aphidicolin treatment, ookinete cultivation *in vitro* was allowed to proceed for 1 hour prior to addition of *Nigrospora sphaerica* aphidicolin at a final concentration of 50 µM (Sigma) [2]. *A. gambiae* Yaoundé and *A. stephensi* sda500 mosquitoes were cultivated and infected with *P. berghei* by either direct feeding on infected mice or ookinete membrane feeding using standard methods. For ookinete hemocoel injections, ookinetes were cultivated for 24 hours and injected into the thorax of *A. stephensi* mosquitoes (800 ookinetes per mosquito) using glass capillary needles and Nanoject II microinjector (Drummond Scientific).

### Ethics statement

Protocols that involved the use of mice were approved by the UK Home Office (Animals Scientific Procedures Act 1986).

### Transcriptional profiling using qRT-PCR or RT-PCR

Total RNA was isolated from parasite stages and mosquito midguts infected with *Δpbmisfit* using the Trizol® reagent (Invitrogen). Gene-specific primers (Table S6) were designed using Primer3 (v. 0.4.0). Quantitative real-time RT-PCR (qRT-PCR) was carried out using SYBR-Green and the ABI Prism 7700 Sequence Detector (Applied Biosystems). *Misfit* transcript levels were normalized against transgenic *gfp* transcripts [53] that provided an internal reference for the fluctuation in parasite numbers during development. Three independent biological replicates were performed, which used different batches of mosquitoes fed on different blood sources (different infected mice). The results of each biological replicate were the average of two technical replicates, in which RNA samples were processed in duplicate in the same qRT-PCR plate. Additional gene-specific primers were used for non-quantitative RT-PCR analysis of *misfit*, *P28*, *ama1*, *cht1*, *ctrp*, *soap*, *maebl* and *warp* (Table S6 and Protocol S1).

### Generation of transgenic parasites

Targeted disruption of *misfit* by double homologous recombination in the *P. berghei* ANKA clone 2.34 or 507 genetic backgrounds was carried out as described [33]. In brief, 816 bp upstream and 870 bp downstream *misfit* target sequences were amplified from *P. berghei* ANKA clone 2.34 genomic DNA using the primer pairs *pbmisfit*A F (*ApaI*)/*pbmisfit*A R (*HindIII*) and *pbmisfit*B F (*EcoRI*)/*pbmisfit*B R (*BamHI*), respectively (Table S6). PCR products were purified using the Promega Wizard® genomic DNA purification kit and cloned into the pBS-DHFR vector that encompasses the *tgdhfr/ts* pyrimethamine resistance cassette. For expression and localization studies, *misfit* was fused to a C-terminal myc tag by replacing 1 kb of the most 3′ terminal portion of the endogenous *misfit* locus with a tagged counterpart. Selection of transgenic parasite lines was carried out by pyrimethamine treatment and limiting dilution cloning to obtain clonal ko lines as described (Protocol S1) [20].

### Genotypic analysis of transgenic parasites

Genomic DNA was prepared from transfected blood-stage parasite populations and subjected to diagnostic PCR and Southern blot analysis to assess successful integration (Protocol S1). For Southern blot analysis of the transgenic lines, genomic DNA was digested with *EcoRV* (*Δpbmisfit*) or *HindIII* (*pbmisfit-myc*). The blot was hybridized against a PCR-generated probe recognizing an 816 bp (*Δpbmisfit*; *pbmisfit* A F and R) or 2 kb (*pbmisfit-myc*; *pbmisfit-myc* F and R) region of *pbmisfit* (Table S6). Pulse field gel electrophoresis was performed on chromosomes derived from purified blood stage parasites as previously described and the blot was hybridized against a probe recognizing the *tgdhfr/ts* cassette obtained by *HindIII* and *EcoRV* double restriction digest of the pBS-DHFR vector.

### Imaging and enumeration of parasites

Blood stage parasite enumeration and monitoring of the quality of preparations of purified gametocytes, male gametes and ookinetes were performed by light microscopy analysis of methanol-fixed Giemsa-stained (Fluka) blood films or parasite smears. Exflagellation assays and macrogamete to ookinete conversion were performed in ookinete medium as described [5]. For oocyst counts and imaging, infected midguts of female *A. stephensi* or *A. gambiae* were dissected in PBS, fixed in 4% formaldehyde in PBS (FA-PBS) for 30–45 min and washed 3 times in PBS for 15 min.

### RNAi gene silencing in mosquitoes

Gene silencing was performed by injections of double stranded RNA in adult mosquitoes as described [22].

### Immunodetection of MISFIT

For Western blot analysis, samples of purified parasites were boiled under reducing conditions in SDS sample loading buffer, prior to 8% SDS-PAGE protein fractionation and immuno-detection according to standard procedures. For immunofluoresence assays (IFA), purified parasite pellets were re-suspended in RPMI∶FCS (1∶1), smeared on glass slides and allowed to air dry prior to fixation in 4% FA-PBS for 10 min. Activated gametocytes were allowed to settle onto poly-L-lysine (0.01%, Sigma) coated glass slides in 4% FA-PBS o/n at 4°C.

MISFIT-MYC was detected using a rabbit anti-myc monoclonal antibody (Mab) (71D10, New England Biolabs) according to manufacturer's instructions at a dilution of 1∶1000 (WB) or 1∶200 (IFA). For co-staining with the rabbit anti-SET antibody (dilution 1∶400) (Pace et al. 2006), MISFIT-myc was detected using a 1∶500 rat anti-myc antibody (JAC6, AbCam). P28 was detected with the 13.1-Cy3 Mab (1∶500). Tubulin I (TUB) was detected with a mouse monoclonal antibody against *Trypanosoma brucei* alpha-tubulin (tat1) at 1∶1000 for immunofluorescence and 1∶10000 for western blot [55]. Secondary antibodies used for IFA at 1∶1500 included: ALEXA FLUOR 488 goat anti-rabbit IgG, ALEXA FLUOR 488 goat anti-rat IgG, ALEXA FLUOR 647 goat anti-mouse or ALEXA FLUOR 647 goat anti-rabbit (Molecular probes). For western blot analysis, horseradish peroxidase (HRP) conjugated goat anti-rabbit IgG (1∶15000) or goat anti-mouse IgG (1∶10000) (Promega) were used.

### DNA quantification

DNA measurements were performed by either DAPI staining and quantification of fluorescent microscopy images, or by DRAQ5 staining and FACS (Fluorescence-Activated Cell Sorting) analysis (Protocol S1).

### Microscopy

Cells or tissues were mounted in VECTASHIELD Mounting Medium with or without DAPI (Vector Labs). Parasites were imaged using a Leica DMT fluorescence microscope and images were captured using a Zeiss AxioCam HRc camera coupled to Zeiss Axiovision40 version 4.6.1.0 software. Post-processing of images was performed using ImageJ 1.40. For IFA, visualization was achieved on a Leica SP5 confocal microscope. Images were background-corrected and noise-filtered with the Leica LAS AF software (Leica Microsystems). 3D projections and additional image adjustments were performed with the Volocity (PerkinElmer Inc) and Adobe Photoshop CS2 (Adobe) software.

### Transmission electron micrographs (TEM)

Ookinetes were pelleted at 3,000 rpm and fixed in 2.5% glutataraldehyde and 2% paraformaldehyde in 0.1 M sodium cacodylate buffer pH 7.42 at RT for 15 min followed by 45 min at 4°C. After rinses, samples were fixed at RT in buffered 1% osmium tetroxide for 1 h followed by mordanting with 1% tannic acid and 1% sodium sulphate and then dehydrated in an ethanol and propylene oxide series, staining *en bloc* with 2% uranyl acetate at the 30% ethanol stage. Samples were embedded in TAAB Araldite 812 resin, ultrathin-sectioned at 60 nm on a Leica EMU6 ultramicrotome, contrasted with uranyl acetate and lead citrate and imaged on a 120 kV FEI Spirit Biotwin with a Tietz TemCam-F415.

### Genetic crosses

Genetic crosses between different ko parasite strains was carried out as described [24] by infecting mice with different combinations of ko parasites. *A. stephensi* mosquitoes were infected by either direct feeding on mice or membrane feeding on ookinetes cultivated *in vitro* from parasites isolated from these mice and re-suspended in naïve mouse blood (800 ookinetes/µl blood). *Δpbnek4*, *Δpbcdpk4*, and *Δpbmap2* parasites were provided by R. Tewari and O. Billker, and *Δpsb47* and *Δpbs48/45* were provided by C.J. Janse and A.P.Waters.

### DNA microarrays

The *Plasmodium* DNA microarray platform used in this study was manufactured by Agilent and encompassed 21,444 oligonucleotide probes for 5,361 *P. berghei* open reading frames [15],[36]. 2 µg of total RNA of *Pbc507 wt* or *Δpbmisfit* ookinetes from three biological experiments were used as templates for the generation of Cy3 or Cy5 CTP (Perkin and Elmer) labeled cRNAs using the Agilent Low RNA Input Fluorescence Amplification Kit Protocol (Protocol S1). After hybridizations and washings, arrays were scanned using a Gene-Pix 4000B scanner and Gene-Pix Pro 4.0 software (Axon instruments). Gene-Pix Pro 6.1 was utilized for grid-alignment, registering spot signal intensity, estimation of local backgrounds and manual inspection of spot quality. Data were subjected to normalization in GeneSpring 6.1 (Agilent) using the locally weighted linear regression method (Lowess) method and analyzed as described [56] using the Cluster software version 2.11 and Java Tree View software version 1.1.0 [57], and Microsoft Excel.

### Phylogenetic analysis

FH2-domain encoding sequences of orthologous MISFIT, Formin1 and Formin2 proteins were aligned separately. The three alignments were sequentially combined using the profile alignment feature implemented in ClustalW. The remaining sequences were individually added to the combined alignment. The phylogenetic tree was built using ClustalW's neighbor-joining algorithm, ignoring all gapped columns and performing 1000 bootstrap samples (branch lengths indicate evolutionary distance).

## Supporting Information

Protocol S1 (0.05 MB DOC)

Table S1 Effect of *misfit* ko on parasite development in *A. stephensi* mosquitoes.
(0.06 MB PDF)

Table S2 Effect of *misfit* disruption on oocyst development.
(0.10 MB PDF)

Table S3 Direct injections of *misfit* ko ookinetes in *A. stephensi* hemocoel.
(0.06 MB PDF)

Table S4 Genetic crosses between *misfit* ko and a panel of female or male gamete deficient *P. berghei* mutants.
(0.09 MB PDF)

Table S5 DNA microarray gene expression data presented as normalized *misfit* ko vs *wt* ookinetes signal intensity ratios. Only genes exhibiting 1.74 (log (2) 0.8) or greater expression between *misfit* ko vs *wt* ookinetes are presented. Results of manual interpro scan analysis are presented (IRP and associated GO terms). PlasmoDB GO analysis is also included. *P. berghei* paralogues as predicted by PlasmoDB as well as ortholoques in other *Plasmodium* species are also shown. Pb, *P. berghei*; Py, *P. yoelli*; Pk, *P. knowlesi*; Pv, *P. vivax*; Pf, *P. falciparum*; orth; orthologues.
(0.12 MB XLS)

Table S6 Primers for qRT-PCR, RT-PCR and generation of *pbmisfit* transgenic parasites.
(0.09 MB PDF)

Figure S1 MISFIT structural features and phylogenetic analysis of the FH2 domain of apicomplexan formin-like proteins. (A) Schematic representation of protein features of PbMISFIT (Pb000064.01.0) and its *P. yoelii* (PyMISFIT; PY00811), *P. falciparum* (PfMISFIT; PF14_0035), *P. knowlesi* (PkMISFIT; PKH_134310, refined annotation) and *P. vivax* (PvMISFIT; Pv086245) orthologues. NLS, nuclear localization signal (red box); FH2, formin homology 2 domain (dark grey box); A putative kinase-like domain (light grey box) with unclassified specificity is predicted for *P. berghei, P. yoelli* and *P. falciparum* MISFITs. NLS was predicted by NucPred (http://www.sbc.su.se/~maccallr/nucpred/) and PredictNLS (http://www.rostlab.org/services/predictNLS/). The C-terminal region of MISFITs (DAD′) resembling the basic region of the Diaphanous-autoregulatory domain (DAD) of DRFs proteins is indicated. NLS sequences are predicted at the same region in *P. knowlesi* and *P. vivax*. A MISFIT orthologue also exists in *Plasmodium chaubaudi*, which due to poor sequence quality is not included in this analysis. (B) Phylogenetic analysis of the FH2 domains of MISFIT, Plasmodium Formin1 and Formin2 and other apicomplexan formin-like proteins. Red and blue circles show 75% and 50% bootstrap support for groups, respectively. PfFormin1 (PFE1545c), PfFormin2 (PFL092w), PvFormin1 (PV079720), PvFormin2 (PV123615), PyFormin1 (PY01292), PyFormin2 (PY01855), TgFormin1 (20.m05986), TgFormin2 (20.m03963), TgFormin3 (31.m00924), TaFormin1 (TA03495), TaFormin2 (TA09030), CpFormin1 (cgd6_4150), CpFormin2 (cgd8_2450), CpFormin3 (cgd8_1500), CpFormin4 (cgd2_3850), Bni1p (NP_014128), AFH1 (NP_189177), DdForA (AB082542), DdForB (ANB082543), DdForC (AB082544), EhFormin1 (XP_653752), EhFormin2 (XP_656030), EhFormin3 (XP_653884), EhFormin4 (XP_651696), EhFormin5 (XP_650406), EhFormin6 (XP_652294), EhFormin7 (XP_653981) and EhFormin8 (XP_650130). Tg, *T. gondii*; Ta, *Theileria annulata*; Cp, *C. parvum*; Sc, *Saccharomyces cerevisiae*; At, *Arabidopsis thaliana*; Dd, *Dictyostelium discoideum*; Eh, *Entamoeba histolytica*.
(0.16 MB PDF)

Figure S2 Multiple sequence alignment of *Plasmodium* MISFIT proteins. Colored blocks indicate amino acid residues conserved among all species. The FH2 domain, a central conserved region and the DAD-like sequence (DAD′) are indicated. Alignment was performed by ClustalW and visualized by BioEdit Sequence Alignment Editor.
(0.15 MB PDF)

Figure S3 Generation of transgenic *Δpbmisfit* parasite. (A) Schematic representation of the pBS-*tgdhfr/ts pbmisfit* disruption vector, and the native and disrupted *pbmisfit* locus. The disruption vector carries pbmisfit targeting sequences, which flanks a *tgdhfr/ts* pyrimethamine-selection cassette. Integration of the *ApaI/BamHI* linerised vector results in the disrupted *pbmisfit* locus. *pbmisfit* coding region (open), *tgdhfr/ts* cassette (grey), predicted transmembrane domains (black). (B) Genotyping of *Δpbmisfit*. The integration of the disruption vector into the pbmisfit locus, held on *P. berghei* c507 chromosome 10, was confirmed by pulse-field gel electrophoresis where the blot was probed with *tgdhfr/ts* fragment. The signal at chromosome 7 derives from cross-hybridization with the native *pbdhfr/ts* locus. (C) PCR based analysis of the genomic DNA from wt and dilution cloned *Δpbmisfit* parasites show that the *Δpbmisfit* locus (*misfit* INT F and *tgdhfr/ts* 5′UTR) is only present in the ko line while the pbmisfit native locus (*misfit* INT and *misfit* WT R) is only present in the Pbc507 *wt* line. (D) Southern blot analysis in which the misfit PCR-amplified fragment (*misfit* A F and R) was used as a probe show that the insertion of the 5 kb *tgdhfr/ts* cassette and simultaneous deletion of 3 kb flanking sequence, resulted in an increase of 2 kb in fragment size of ko compared to the *wt* locus.
(0.65 MB PDF)

Figure S4 Construction of *pbmisfit-myc* transgenic parasite. (A) Schematic representation of the pBS-tgdhfr/ts *pbmisfit*-myc-tagging vector, the native *pbmisfit* locus and the resulting transgenic *pbmisfit-myc* locus. The tagging vector carries a C-terminal fragment of *pbmisfit*, with a unique *ClaI* restriction site in its centre, cloned in-frame with a double c-myc tag (black) and in tandem with a *tgdhfr/ts* pyrimethamine-selection cassette. Following construct linearization with *ClaI*, the *pbmisfit-myc* sequence is separated into two fragments, *misfit ^a[c]^* and *misfit ^b[c]^-myc*, where superscript *[c]* stands for cassette. Transfection of the linearized cassette on *P. berghei* (ANKA 2.34 strain) results in single homologous recombination and replacement of the last 942 bp of the native *pbmisfit* locus (*misfit ^b[wt]^*) with its myc-tagged version (*misfit ^b[c]^*-myc). (B) Genotyping of *pbmisfit-myc*. The integration of the tagging vector into the native *pbmisfit* locus in chromosome 10 was confirmed by pulse-field gel electrophoresis using the *tgdhfr/ts* fragment as probe. The signal at chromosome 7 derives from cross-hybridization with the native *pbdhfr/ts* locus.
(0.59 MB PDF)

Figure S5 Phenotypic analysis and invasion assays of Aphidicolin treated ookinetes. (A) Fluorescent microscopy images of 7-day old GFP-expressing oocysts of *wt*, *Δpbmisfit* and *wt* Aphidicolin-treated parasites in *A. gambiae* midguts. (B) Microscopy images of *wt*, *Δpbmisfit* and Aphidicolin-treated ookinetes that are melanized immediately after invasion of CTL4 kd *A. gambiae* midguts. Aphidicolin treated ookinetes, like *Δpbmisfit*, do invade the midgut but fail to produce sporulating oocysts. Insets show oocysts and melanized parasites in high magnification.
(3.92 MB PDF)

## References

[1] Janse CJ, Boorsma EG, Ramesar J, van Vianen P, van der Meer R (1989). Plasmodium berghei: gametocyte production, DNA content, and chromosome-size polymorphisms during asexual multiplication in vivo.. Exp Parasitol.

[2] Janse CJ, Van der Klooster PF, Van der Kaay HJ, Van der Ploeg M, Overdulve JP (1986). Rapid repeated DNA replication during microgametogenesis and DNA synthesis in young zygotes of Plasmodium berghei.. Trans R Soc Trop Med Hyg.

[3] Sinden RE (1999). Plasmodium differentiation in the mosquito.. Parassitologia.

[4] Sinden RE, Strong K (1978). An ultrastructural study of the sporogonic development of Plasmodium falciparum in Anopheles gambiae.. Trans R Soc Trop Med Hyg.

[5] Billker O, Dechamps S, Tewari R, Wenig G, Franke-Fayard B (2004). Calcium and a calcium-dependent protein kinase regulate gamete formation and mosquito transmission in a malaria parasite.. Cell.

[6] Khan SM, Franke-Fayard B, Mair GR, Lasonder E, Janse CJ (2005). Proteome analysis of separated male and female gametocytes reveals novel sex-specific Plasmodium biology.. Cell.

[7] Tewari R, Dorin D, Moon R, Doerig C, Billker O (2005). An atypical mitogen-activated protein kinase controls cytokinesis and flagellar motility during male gamete formation in a malaria parasite.. Mol Microbiol.

[8] Sinden RE, Hartley RH (1985). Identification of the meiotic division of malarial parasites.. J Protozool.

[9] van Dijk MR, Janse CJ, Thompson J, Waters AP, Braks JA (2001). A central role for P48/45 in malaria parasite male gamete fertility.. Cell.

[10] Liu Y, Tewari R, Ning J, Blagborough AM, Garbom S (2008). The conserved plant sterility gene HAP2 functions after attachment of fusogenic membranes in Chlamydomonas and Plasmodium gametes.. Genes Dev.

[11] Hirai M, Arai M, Mori T, Miyagishima SY, Kawai S (2008). Male fertility of malaria parasites is determined by GCS1, a plant-type reproduction factor.. Curr Biol.

[12] Reininger L, Billker O, Tewari R, Mukhopadhyay A, Fennell C (2005). A NIMA-related protein kinase is essential for completion of the sexual cycle of malaria parasites.. J Biol Chem.

[13] Canning EU, Sinden RE (1973). The organization of the ookinete and observations on nuclear division in oocysts of Plasmodium berghei.. Parasitology.

[14] Wallar BJ, Alberts AS (2003). The formins: active scaffolds that remodel the cytoskeleton.. Trends Cell Biol.

[15] Hall N, Karras M, Raine JD, Carlton JM, Kooij TW (2005). A comprehensive survey of the Plasmodium life cycle by genomic, transcriptomic, and proteomic analyses.. Science.

[16] Doerks T, Copley RR, Schultz J, Ponting CP, Bork P (2002). Systematic identification of novel protein domain families associated with nuclear functions.. Genome Res.

[17] Wallar BJ, Stropich BN, Schoenherr JA, Holman HA, Kitchen SM (2006). The basic region of the diaphanous-autoregulatory domain (DAD) is required for autoregulatory interactions with the diaphanous-related formin inhibitory domain.. J Biol Chem.

[18] Baum J, Tonkin CJ, Paul AS, Rug M, Smith BJ (2008). A malaria parasite formin regulates actin polymerization and localizes to the parasite-erythrocyte moving junction during invasion.. Cell Host Microbe.

[19] Schuler H, Matuschewski K (2006). Regulation of apicomplexan microfilament dynamics by a minimal set of actin-binding proteins.. Traffic.

[20] Janse CJ, Franke-Fayard B, Mair GR, Ramesar J, Thiel C (2006). High efficiency transfection of Plasmodium berghei facilitates novel selection procedures.. Mol Biochem Parasitol.

[21] Paton MG, Barker GC, Matsuoka H, Ramesar J, Janse CJ (1993). Structure and expression of a post-transcriptionally regulated malaria gene encoding a surface protein from the sexual stages of Plasmodium berghei.. Mol Biochem Parasitol.

[22] Osta MA, Christophides GK, Kafatos FC (2004). Effects of mosquito genes on Plasmodium development.. Science.

[23] Pace T, Olivieri A, Sanchez M, Albanesi V, Picci L (2006). Set regulation in asexual and sexual Plasmodium parasites reveals a novel mechanism of stage-specific expression.. Mol Microbiol.

[24] Raine JD, Ecker A, Mendoza J, Tewari R, Stanway RR (2007). Female inheritance of malarial lap genes is essential for mosquito transmission.. PLoS Pathog.

[25] Ecker A, Pinto SB, Baker KW, Kafatos FC, Sinden RE (2007). Plasmodium berghei: plasmodium perforin-like protein 5 is required for mosquito midgut invasion in Anopheles stephensi.. Exp Parasitol.

[26] Ecker A, Bushell ES, Tewari R, Sinden RE (2008). Reverse genetics screen identifies six proteins important for malaria development in the mosquito.. Mol Microbiol.

[27] Gissot M, Briquet S, Refour P, Boschet C, Vaquero C (2005). PfMyb1, a Plasmodium falciparum transcription factor, is required for intra-erythrocytic growth and controls key genes for cell cycle regulation.. J Mol Biol.

[28] Hadjebi O, Casas-Terradellas E, Garcia-Gonzalo FR, Rosa JL (2008). The RCC1 superfamily: from genes, to function, to disease.. Biochim Biophys Acta.

[29] Gissot M, Ting LM, Daly TM, Bergman LW, Sinnis P (2008). High mobility group protein HMGB2 is a critical regulator of plasmodium oocyst development.. J Biol Chem.

[30] Dessens JT, Mendoza J, Claudianos C, Vinetz JM, Khater E (2001). Knockout of the rodent malaria parasite chitinase pbCHT1 reduces infectivity to mosquitoes.. Infect Immun.

[31] Langer RC, Vinetz JM (2001). Plasmodium ookinete-secreted chitinase and parasite penetration of the mosquito peritrophic matrix.. Trends Parasitol.

[32] Yuda M, Yano K, Tsuboi T, Torii M, Chinzei Y (2001). von Willebrand Factor A domain-related protein, a novel microneme protein of the malaria ookinete highly conserved throughout Plasmodium parasites.. Mol Biochem Parasitol.

[33] Dessens JT, Beetsma AL, Dimopoulos G, Wengelnik K, Crisanti A (1999). CTRP is essential for mosquito infection by malaria ookinetes.. Embo J.

[34] Yuda M, Sakaida H, Chinzei Y (1999). Targeted disruption of the plasmodium berghei CTRP gene reveals its essential role in malaria infection of the vector mosquito.. J Exp Med.

[35] Dessens JT, Siden-Kiamos I, Mendoza J, Mahairaki V, Khater E (2003). SOAP, a novel malaria ookinete protein involved in mosquito midgut invasion and oocyst development.. Mol Microbiol.

[36] Mair GR, Braks JA, Garver LS, Wiegant JC, Hall N (2006). Regulation of sexual development of Plasmodium by translational repression.. Science.

[37] Lavazec C, Moreira CK, Mair GR, Waters AP, Janse CJ (2009). Analysis of mutant Plasmodium berghei parasites lacking expression of multiple PbCCp genes.. Mol Biochem Parasitol.

[38] Scherf A, Lopez-Rubio JJ, Riviere L (2008). Antigenic variation in Plasmodium falciparum.. Annu Rev Microbiol.

[39] Hakimi MA, Deitsch KW (2007). Epigenetics in Apicomplexa: control of gene expression during cell cycle progression, differentiation and antigenic variation.. Curr Opin Microbiol.

[40] Higgs HN, Peterson KJ (2005). Phylogenetic analysis of the formin homology 2 domain.. Mol Biol Cell.

[41] Bartolini F, Moseley JB, Schmoranzer J, Cassimeris L, Goode BL (2008). The formin mDia2 stabilizes microtubules independently of its actin nucleation activity.. J Cell Biol.

[42] Gomez TS, Kumar K, Medeiros RB, Shimizu Y, Leibson PJ (2007). Formins regulate the actin-related protein 2/3 complex-independent polarization of the centrosome to the immunological synapse.. Immunity.

[43] Kato T, Watanabe N, Morishima Y, Fujita A, Ishizaki T (2001). Localization of a mammalian homolog of diaphanous, mDia1, to the mitotic spindle in HeLa cells.. J Cell Sci.

[44] Leader B, Lim H, Carabatsos MJ, Harrington A, Ecsedy J (2002). Formin-2, polyploidy, hypofertility and positioning of the meiotic spindle in mouse oocytes.. Nat Cell Biol.

[45] Majumder S, Lohia A (2008). Entamoeba histolytica encodes unique formins, a subset of which regulates DNA content and cell division.. Infect Immun.

[46] Doerig C, Chakrabarti D, Waters AP, Janse CJ (2004). Cell cycle control in Plasmodium falciparum: A genomics perspective.. Malaria parasites: Genomes and molecular biology.

[47] Sinden RE, Killick-Kendrick R, Peters WA (1978). Cell Biology.. Rodent Malaria.

[48] Miki T, Okawa K, Sekimoto T, Yoneda Y, Watanabe S (2009). mDia2 Shuttles between the Nucleus and the Cytoplasm through the Importin-{alpha}/{beta}- and CRM1-mediated Nuclear Transport Mechanism.. J Biol Chem.

[49] Gubbels MJ, Lehmann M, Muthalagi M, Jerome ME, Brooks CF (2008). Forward genetic analysis of the apicomplexan cell division cycle in Toxoplasma gondii.. PLoS Pathog.

[50] Blackman MJ, Bannister LH (2001). Apical organelles of Apicomplexa: biology and isolation by subcellular fractionation.. Mol Biochem Parasitol.

[51] Schrevel J, Asfaux-Foucher G, Hopkins JM, Robert V, Bourgouin C (2008). Vesicle trafficking during sporozoite development in Plasmodium berghei: ultrastructural evidence for a novel trafficking mechanism.. Parasitology.

[52] de Koning-Ward TF, Olivieri A, Bertuccini L, Hood A, Silvestrini F (2008). The role of osmiophilic bodies and Pfg377 expression in female gametocyte emergence and mosquito infectivity in the human malaria parasite Plasmodium falciparum.. Mol Microbiol.

[53] Franke-Fayard B, Trueman H, Ramesar J, Mendoza J, van der Keur M (2004). A Plasmodium berghei reference line that constitutively expresses GFP at a high level throughout the complete life cycle.. Mol Biochem Parasitol.

[54] Carter R, Chen DH (1976). Malaria transmission blocked by immunisation with gametes of the malaria parasite.. Nature.

[55] Woods A, Sherwin T, Sasse R, MacRae TH, Baines AJ (1989). Definition of individual components within the cytoskeleton of Trypanosoma brucei by a library of monoclonal antibodies.. J Cell Sci.

[56] Vlachou D, Schlegelmilch T, Christophides GK, Kafatos FC (2005). Functional genomic analysis of midgut epithelial responses in Anopheles during Plasmodium invasion.. Curr Biol.

[57] Eisen MB, Spellman PT, Brown PO, Botstein D (1998). Cluster analysis and display of genome-wide expression patterns.. Proc Natl Acad Sci U S A.

