# Supplementary material for: Paternal Effect of the Nuclear Formin-like Protein MISFIT on Plasmodium Development in the Mosquito Vector
Source: PLoS Pathog. 2009 Aug 7;5(8):e1000539. doi: 10.1371/journal.ppat.1000539 (PMC2715856; doi:10.1371/journal.ppat.1000539)
Supplement: Protocol S1 — (0.05 MB DOC) [file ppat.1000539.s001.doc]

**Paternal Effect of the Nuclear Formin-like Protein MISFIT on *Plasmodium* Development in the Mosquito Vector**

**Ellen S. C. Bushell, Andrea Ecker, Timm Schlegelmilch, David Goulding, Gordon Dougan, Robert E. Sinden, George K. Christophides, Fotis C. Kafatos, Dina Vlachou**

**Protocol S1**

Parasite purification

Purifications of asexual and sexual blood stage parasites and male microgametes were performed as described [1,2,3]. For preparation of pure viable gametocytes, sulphadiazine (Sigma) treatment was used in conjunction with Nycodenz (Life technologies) density gradient purification. Purified gametocytes were collected from the gradient interface, and harvested immediately or following activation by incubation in ookinete culture medium at 19ºC. Gametocyte viability was tested before and after purification by microscopic observations of exflagellation in ookinete medium. Ookinete purification was facilitated by utilizing sheep anti-mouse IgG Dynal Dynabeads (Invitrogen) coated in a P28 mouse monoclonal antibody (13.1) as described [4].

Transcriptional profiling using RT-PCR or qRT-PCR

For *in vivo* mosquito infections, Pb29c12 *wt* infected midguts were dissected on ice in PBS. Mixed blood stages, gametocytes and ookinetes of Pb507 *wt* and *Δpbmisfit* were purified as described above. Tissues or cells were snap-frozen in liquid nitrogen and stored at -80ºC. Following mechanical homogenization in Trizol® reagent (Invitrogen), total RNA was isolated according to manufacturer’s instructions and samples were DNAseI treated (New England Biolabs) for 15 min at 37ºC and purified using RNAeasy columns (Qiagen). The quality and quantity of RNA were determined by standard gel electrophoresis and spectrophotometry using the nucleic acid application of NanoDrop® ND-1000 spectrophotometer. Reverse transcription of mRNA (1μg total RNA) was performed using a combination of random hexamers and oligo-dT primers (TaqMan reverse Transcription Reagents Kit; Applied Biosystems).

Quantitative real-time RT-PCR (qRT-PCR) was conducted using the primers *misfit* QRT-PCR F at 200nM and R at 900nM (Table S6), SYBR-Green detection and amplification reagents (Applied Biosystems), and ABI PRISM 7000 sequence detector (Applied Biosystems). All reactions were run in duplicates and the final transcriptional profile was generated from the average of three biological experiments. *Misfit* transcript levels were normalized against *gfp* transcription (*gfp* qRT-PCR F at 900nM and R at 200nM; Table S6), which is driven by the elongation factor-1alpha promoter [5] and hence provides an internal reference for the fluctuation in parasite numbers during development. For non-quantitative or semi-quantitative RT-PCR (RT-PCR), a second set of primers (*misfit* RT-PCR F and R, Table S6) was used, which generates a 1 kb amplicon. Additional gene-specific primers were designed for RT-PCR analysis of *p28*, *ama1*, *chit1*, *ctrp*, *maebl*, *soap* and *warp* (Table S6). The PCR program used for RT-PCR comprised of 5 min at 94ºC, followed by 20, 25 or 30 cycles of 30 sec at 94ºC, 30 sec at 52ºC and 1 min at 62ºC, completed by 10 min at 62ºC. PCR-products were visualized by standard gel electrophoresis.

Generation of transgenic parasites

Targeted disruption of *pbmisfit* by double homologous recombination was carried out as described [2]. In brief, 816 bp upstream and 870 bp downstream *misfit* target sequences were amplified from *P. berghei* ANKA clone 2.34 genomic DNA using the primer pairs *misfit* A F (*ApaI*) / *misfit* A R (*HindII*) and *misfit* B F (*EcoRI*) / *misfit* B R (*BamHI*), respectively (Table S6). PCR products were purified using the Promega Wizard® genomic DNA purification kit and cloned into the pBS-DHFR vector, in which polylinker sites flank a *tgdhfr/ts* pyrimethamine resistance cassette [2]. The targeting cassette was released from the vector by *ApaI* and *BamHI* double restriction digest.

For expression and localization studies, *pbmisfit* was fused to a c-terminal myc tag by replacing 1 kb of the most 3′ terminal portion of the endogenous *pbmisfit* locus with a tagged counterpart. The tagging construct was engineered by cloning a PCR-amplified 2.0 kb *pbmisfit* targeting region (*misfit*-myc F (*KpnI*) and R (*ApaI*) primers, (Table S6) in-frame with a c-terminal myc-tag held in the pDR0007 tagging vector, which also carries a *tgdhfr/ts* selection cassette (kind gift from D. J. Raine). The tagging construct was sequenced (*misfit*-myc seq F primers 1 and 2; Table S6) and linearized by *ClaI* digestion, which cuts 1kb into the *pbmisfit* targeting region, allowing for introduction of the myc-tag vector by single homologous crossover. Linear constructs were purified by ethanol precipitation, and 3-5 μg of DNA in a volume of 5-10 μl were used for transfections using the Human T-Cell Nucleofector Kit (AMAXA). Selection of transgenic parasite lines by pyrimethamine treatment and limiting dilution cloning to obtain clonal KO lines were carried out as described [6]. Transgenic parasite lines were generated in two separate genetic backgrounds, *P. berghei* ANKA clone 2.34 or 507.

Genotypic analysis of transgenic parasites

Genomic DNA was prepared from transfected blood-stage parasite populations and subjected to diagnostic PCR and Southern blot analysis to assess successful integration. For targeted disruption, PCR based analysis of the *Δpbmisfit* locus was performed by using a forward primer (*misfit* A INT-F) in combination with the *tgdhfr/ts* 5'UTR R primer (Table S6). The primer positioning ensures that a product can only be obtained if integration of the disruption cassette has occurred. Absence of the *pbmisfit wt* locus was verified by the use of *misfit* INT F primer in combination with the *misfit* WT R primer (Table S6) located within the region of the *wt* locus that has been excised by homologous recombination in the *Δpbmisfit* parasite. For Southern blot analysis of the transgenic lines, genomic DNA was digested with *EcoRV* (*Δpbmisfit*) or *HindIII* (*pbmisfit-myc*). The blot was hybridized against a PCR-generated probe recognizing an 816 bp (Δ*pbmisfit*; *misfit* A F and R) or 2 kb (*pbmisfit-myc*; *misfit-myc* F and R) region of *pbmisfit*. Pulse field gel electrophoresis was performed on chromosomes derived from purified blood stage parasites as previously described [7] and the blot was hybridized against a probe recognizing the *tgdhfr/ts* cassette. The *tgdhfr/ts* probe fragment was obtained by *HindIII* and *EcoRV* double restriction digest of the pBS-DHFR vector.

Imaging and enumeration of parasites

Blood stage parasite enumeration, and monitoring of the quality of preparations of purified gametocytes, male gametes and ookinetes was performed by light microscopy analysis of methanol-fixed, Giemsa (Fluka) stained blood films or purified parasite smears. Exflagellation assays and macrogamete to ookinete conversion assays (utilizing a Cy3 conjugated P28 (13.1) monoclonal antibody) was performed in ookinete medium as described [8]. For oocyst counts and imaging, infected midguts of female *A. stephensi* or *A. gambiae* were dissected in PBS, fixed in 4% formaldehyde (BDH) PBS for 30-45 min and washed 3 times in PBS for 15 min.

DNA quantification

Ookinetes for DNA quantification were cultivated for 24 hours, prepared and fixed as described for IFA. Following fixation, the cells were washed three times in PBS for 10 min at r/t, where the second wash was supplemented with DAPI (1/2000, Boehringer Mannheim GmbH). Images for DNA measurements were captured using a fluorescence microscope with a fixed exposure time. Image processing was performed using the ImageJ software version 1.40 (National Institute for Health). The relative nuclear DAPI intensity was calculated as the function of the total number of pixels and the average pixel intensity. Ookinete nuclear DAPI intensity was normalized against the haploid value of ring stage and early trophozoites placed on the same slide. ANOVA statistical analysis was performed. DNA quantification of *wt*, *misfit* ko and ahphidicolin-treated *wt* parasites were also performed by FACS (Fluorescence-Activated Cell Sorting) analysis. Briefly, *in vitro* producing ookinetes were stained with DRAQ5^TM^ according to manufacturer’s instructions and DRAQ5^TM^ fluorescence intensity was determined by flow cytometry.

Microscopy

Cells or tissue were mounted in VECTASHIELD Mounting Medium with or without DAPI (Vector Labs). Parasites were imaged using a Leica DMT fluorescence microscope and images captured using a Zeiss AxioCam HRc camera coupled to Zeiss Axiovision40 version 4.6.1.0 software. Post-processing of images was performed using ImageJ 1.40. For IFA, visualization was achieved on a Leica SP5 confocal microscope. Images were background-corrected and noise-filtered with the Leica LAS AF software (Leica Microsystems). 3D projections and additional image adjustments were performed with the Volocity (PerkinElmer Inc) and Adobe Photoshop CS2 (Adobe) software packages.

DNA microarrays

2 μg of total RNA was used as template for the generation of Cy3 or Cy5 CTP (Perkin and Elmer) labeled cRNA using the Agilent Low RNA Input Fluorescence Amplification Kit Protocol. Labeled cRNA was purified using Qiagen RNAeasy purification columns and its concentration and dye-incorporation was measured using the microarray application of NanoDrop® ND-1000 Spectrophotometer. 2 μg of *Pbc507 wt* (Cy5) and *Δpbmisfit* (Cy3) labeled cRNA (10-20 pg Cy-dye/ μg cRNA) were fragmented, mixed with hybridization solution to a final volume of 80 μl and applied to the microarray. Microarrays were manufactured by Agilent and encompassed 21,444 oligonucleotide probes for 5,361 *P. berghei* open reading frames [9,10]. Hybridization reactions were incubated at 60ºC for 17 hours in a GeneMachine HybChamber™. Following hybridization, slides were washed at RT for 1 min in 6X SSC and 0.005% Triton X-102, followed by 1 min in 0.1X SSC and 0.005% Triton X-102, dried using compressed air and stored under vacuum in the dark.

**Supporting References**

1. Carter R, Chen DH (1976) Malaria transmission blocked by immunisation with gametes of the malaria parasite. Nature 263: 57-60.

2. Dessens JT, Beetsma AL, Dimopoulos G, Wengelnik K, Crisanti A, et al. (1999) CTRP is essential for mosquito infection by malaria ookinetes. Embo J 18: 6221-6227.

3. Liu Y, Tewari R, Ning J, Blagborough AM, Garbom S, et al. (2008) The conserved plant sterility gene HAP2 functions after attachment of fusogenic membranes in Chlamydomonas and Plasmodium gametes. Genes Dev 22: 1051-1068.

4. Siden-Kiamos I, Vlachou D, Margos G, Beetsma A, Waters AP, et al. (2000) Distinct roles for pbs21 and pbs25 in the in vitro ookinete to oocyst transformation of Plasmodium berghei. J Cell Sci 113 Pt 19: 3419-3426.

5. Franke-Fayard B, Trueman H, Ramesar J, Mendoza J, van der Keur M, et al. (2004) A Plasmodium berghei reference line that constitutively expresses GFP at a high level throughout the complete life cycle. Mol Biochem Parasitol 137: 23-33.

6. Janse CJ, Franke-Fayard B, Mair GR, Ramesar J, Thiel C, et al. (2006) High efficiency transfection of Plasmodium berghei facilitates novel selection procedures. Mol Biochem Parasitol 145: 60-70.

7. Reininger L, Billker O, Tewari R, Mukhopadhyay A, Fennell C, et al. (2005) A NIMA-related protein kinase is essential for completion of the sexual cycle of malaria parasites. J Biol Chem 280: 31957-31964.

8. Billker O, Dechamps S, Tewari R, Wenig G, Franke-Fayard B, et al. (2004) Calcium and a calcium-dependent protein kinase regulate gamete formation and mosquito transmission in a malaria parasite. Cell 117: 503-514.

9. Hall N, Karras M, Raine JD, Carlton JM, Kooij TW, et al. (2005) A comprehensive survey of the Plasmodium life cycle by genomic, transcriptomic, and proteomic analyses. Science 307: 82-86.

10. Mair GR, Braks JA, Garver LS, Wiegant JC, Hall N, et al. (2006) Regulation of sexual development of Plasmodium by translational repression. Science 313: 667-669.
