# Supplementary material for: Paternal Effect of the Nuclear Formin-like Protein MISFIT on Plasmodium Development in the Mosquito Vector
Source: PLoS Pathog. 2009 Aug 7;5(8):e1000539. doi: 10.1371/journal.ppat.1000539 (PMC2715856; doi:10.1371/journal.ppat.1000539)
Supplement: Table S1 — Effect of misfit ko on parasite development in A. stephensi mosquitoes. (0.06 MB PDF) [file ppat.1000539.s002.pdf]

Table S1. Effect of *misfit* ko on parasite development in *A. stephensi* mosquitoes.

Parasite	Midgut sporozoites		Salivary gland sporozoites		Infectivity to mice	
	Mean	SE	Mean	SE	Day 18	Day 21
wt	59,463	2,572	6,762	3,372	2	2
	98,391	1,639	9,513	1,779	2	2
	48,165	7,151	22,978	8,986	2	2
Δpbmisfit	0	0	0	0	0	0
	82	82	0	0	0	0
	54	48	0	0	0	0

The table outlines mean Pbc507 *wt* or *Δpbmisfit* sporozoite densities in *A. stephensi* midguts and salivary glands. The mean was calculated by quantifying the number of sporozoites in suspensions from three pools of ten homogenised midguts or salivary glands, respectively, at day 21 post infection. SE represents standard error. Infectivity to mice was assayed by allowing *wt* or *Δpbmisfit* infected mosquitoes to feed on two C57BL/6 mice (bite-back) at day 18 and 21 of infection, respectively. Mice were allowed to recover and parasitaemia was assessed at day 5 post-feeding and up to day 14 if infection was not detected. Three independent paired infections were performed and reported. The development of oocysts in a small number of mosquitoes at day 15 post-feeding was monitored to assess the success of each infection.
