# Supplementary material for: Paternal Effect of the Nuclear Formin-like Protein MISFIT on Plasmodium Development in the Mosquito Vector
Source: PLoS Pathog. 2009 Aug 7;5(8):e1000539. doi: 10.1371/journal.ppat.1000539 (PMC2715856; doi:10.1371/journal.ppat.1000539)
Supplement: Table S2 — Effect of misfit disruption on oocyst development. (0.10 MB PDF) [file ppat.1000539.s003.pdf]

Table S2. Effect of *misfit* disruption on oocyst development.

Time point	Parasite	Number of exp	Number of midguts	Prevalence (%)	Parasite intensity		Parasite range	Analysis of variance		Fold difference
					Arithmetic mean	Gmean		P value	F value	
***A. gambiae* infection**										
Day 3	*wt*	2	36	83.3	41.8	10.0	0-412	<0.001	16.7	5.8
	Δpbmisfit			58.3	4.0	1.7	0-25			
Day 5	*wt*	2	28	85.7	54.5	20.2	0-251	<0.001	30.7	9.8
	Δpbmisfit			75.0	5.1*	2.0	0-74			
Day 10	*wt*	2	63	94.0	52.0	22.2	0-309	<0.001	126.2	18.9
	Δpbmisfit			47.6	3.4*	1.2	0-60			
Day 15	*wt*	2	39	84.6	23.1	10.0	0-149	<0.001	62.2	20.0
	Δpbmisfit			30.8	1.0*	0.5	0-6			
***A. stephensi* infection**										
Day 3	*wt*	2	27	96.3	377.8	290.7	0-580	<0.001	32.5	5.3
	Δpbmisfit			92.6	117.3	54.6	0-400			
Day 5	*wt*	2	32	96.9	304.0	194.5	0-620	<0.001	17.6	4.4
	Δpbmisfit			93.8	77.6*	44.4	0-244			
Day 10	*wt*	2	66	100	356.8	306.3	0-750	<0.001	169.3	8.4
	Δpbmisfit			100	61.3*	36.4	0-344			
Day 15	*wt*	2	41	100	318.6	243.2	0-670	<0.001	254.4	33.9
	Δpbmisfit			100	12.9*	7.2	0-110			
***CTL4* kd *A. gambiae* infection (ookinete invasion assay)**										
Day 7	*wt*	2	44	75	141.6	19.9	0-632	0.970	0.0	No difference
	Δpbmisfit		44	84.1	95.3	20.1	0-616			

The table is divided into three datasets (separated by dotted lines) that report results from *Δpbmisfit* or Pbc507 *wt* parasite infections of *A. gambiae*, *A. stephensi*, and *A. gambiae CTL4* kd (for ookinete invasion assay), respectively. The first two datasets report numbers of oocysts and the third dataset reports numbers of melanized parasites. Equal numbers of *Δpbmisfit* or *wt* infected midguts (indicated in the second column) were pooled for each group. The total number of midguts is indicated in the third column. Prevalence shows the percentage of midguts with at least one oocyst or melanized parasite. The oocyst intensity data (average number of oocysts per midgut) were log-transformed [$\log_{10}(n+1)$] to achieve a norm distribution. Midguts with zero parasites were also considered for calculation of the arithmetic and geometric (following data normalization) means of parasite densities (number per midgut). P and F one-way ANOVA values for each *Δpbmisfit* vs. *wt* infection were calculated using normalized intensities. In *CTL4* kd infections, the intensity data refers to the number of melanised ookinetes. Fold differences between *wt* and *Δpbmisfit* oocyst intensities were computed using the geometric means. Asterisks indicate small and non-sporulating *Δpbmisfit* oocysts.
