# Supplementary material for: Paternal Effect of the Nuclear Formin-like Protein MISFIT on Plasmodium Development in the Mosquito Vector
Source: PLoS Pathog. 2009 Aug 7;5(8):e1000539. doi: 10.1371/journal.ppat.1000539 (PMC2715856; doi:10.1371/journal.ppat.1000539)
Supplement: Table S3 — Direct injections of misfit ko ookinetes in A. stephensi hemocoel. (0.06 MB PDF) [file ppat.1000539.s004.pdf]

Table S3. Direct injections of *misfit* ko ookinetes in *A. stephensi* hemocoel

Parasite	Salivary gland sporozoites		Infectivity to mice
	Arithmetic Mean	SE	
Pb507 *wt*	1027.5	727.5	2
	15258.8	N/A	2
	6077.5	3442.5	2
Δpbmisfit	0	0	0
	0	0	0
	0	0	0

Results from Pbc507 *wt* or *Δpbmisfit* ookinete injections in *A. stephensi* hemocoel. The readout were salivary gland sporozoites at day 21 post-injection given as their arithmetic mean per mosquito (quantified in suspensions of 20 homogenised salivary glands, assayed in two batches of ten) and infectivity to mice when these mosquitoes were allowed to bite and feed on C57/BL6 mice 21 days post injection (bite-back assays). In bite-back assays, each pot of mosquitoes fed on two mice. The mice blood-stage parasitaemia was assessed at day 5 post-feeding or up to day 14 if mice were found not infected.
