# Supplementary material for: Paternal Effect of the Nuclear Formin-like Protein MISFIT on Plasmodium Development in the Mosquito Vector
Source: PLoS Pathog. 2009 Aug 7;5(8):e1000539. doi: 10.1371/journal.ppat.1000539 (PMC2715856; doi:10.1371/journal.ppat.1000539)
Supplement: Table S4 — Genetic crosses between misfit ko and a panel of female or male gamete deficient P. berghei mutants. (0.09 MB PDF) [file ppat.1000539.s005.pdf]

Table S4. Genetic crosses between *misfit* ko and a panel of female or male gamete deficient *P. berghei* mutants

Genetic crosses	Oocyst densities	Prevalence	Oocyst size	Sporulation	# of midguts	Type of feed
Δpbmisfit in GFP reference line genetic background						
Δpbmisfit (♀) x *Δpbs47* (♂)	62	100%	Normal	√	20	Membrane
	53	100%	Normal	√	40	Membrane
	13	100%	Normal	√	12	Membrane
	179	100%	Normal	√	45	Direct
Δpbmisfit (♂) x *Δpbs48/45* (♀)	22	43%	Small	–	30	Membrane
	9	16%	Small	–	44	Membrane
	21	40%	Small	–	10	Membrane
Δpbmisfit (♂) x *Δpbcdpk4* (♀)	19	83%	Small	–	29	Direct
	21	92%	Small	–	51	Direct
Δpbmisfit (♂) x *Δpbmap2* (♀)	18	100%	Small	–	58	Direct
	31	100%	Small	–	15	Direct
Δpbmisfit in ANKA 2.34 *wt* genetic background						
Δpbmisfit (♀) x *Δpbs47* (♂)	97	100%	Normal	√	45	Membrane
Δpbmisfit (♂) x *Δpbs48/45* (♀)	1	37%	Small	–	22	Membrane
Δpbmisfit (♂) x *Δpbcdpk4* (♀)	7	83%	Small	–	47	Direct
	3.1	82%	Small	–	50	Direct
Δpbmisfit (♀) x *Δpbnek4* (♂)	34	96%	Normal	√	49	Direct
	213	100%	Normal	√	50	Direct
Control crosses of *Δpbpplp5*						
Δpplp5 (♂) x *Δpbcdpk4* (♀)	30	96%	Normal	√	50	Direct
Δpplp5 (♂) x *Δpbmap2* (♀)	39	86%	Normal	√	50	Direct

The table reports the outcome of *A. stephensi* infections with *P. berghei* parasites generated by genetically crossing *Δpbmisfit* (*misfit* ko) parasites with mutants that are either female (*Δpbs47*, *Δpbnek4*) or male (*Δpbs48/45*, *Δpbcdpk4*, *Δpbmap2*) gamete deficient. The results from two independent *misfit* ko lines, where *misfit* was disrupted in the Pbc507 (GFP reference) or ANKA 2.34 *wt* genetic background lines, respectively, are shown. Crosses between the male gamete deficient *Δpbcdpk4* or *Δpbmap2* and *Δpplp5* mutants were used as a control. Each line represents an independent crossing and infection experiment, carried out by feeding mosquitoes either via a membrane on ookinete *in vitro* cultures initiated with blood of mice co-infected with the indicated mutants or directly on co-infected mice. Only crosses where males carry functional *misfit* alleles result in normal oocyst development. Crosses where functional *misfit* alleles are provided only by female gametes result in oocysts of reduced prevalence (percentage of midguts with at least one oocyst) or density (mean oocyst number per infected midgut), small in size and which fail to sporulate.
