# Supplementary material for: Paternal Effect of the Nuclear Formin-like Protein MISFIT on Plasmodium Development in the Mosquito Vector
Source: PLoS Pathog. 2009 Aug 7;5(8):e1000539. doi: 10.1371/journal.ppat.1000539 (PMC2715856; doi:10.1371/journal.ppat.1000539)
Supplement: Table S6 — Primers for qRT-PCR, RT-PCR and generation of pbmisfit transgenic parasites. (0.09 MB PDF) [file ppat.1000539.s007.pdf]

Table S6. Primers for qRT-PCR, RT-PCR and generation of *pbmisfit* transgenic parasites

Primer name	Sequence (5′ to 3′)	Description
misfit QRT-PCR F	CAAATGATTCGGAAATGTTGC	
misfit QRT-PCR R	CAATTTGCTATCATCGCCCT	
misfit RT-PCR F	GATGAAGAAAAGGACAAAGAAATTTC	
misfit RT-PCR R	AAGGGATACATATTCACAATCAAG	
gfp QRT-PCR F	CCTGTCCTTTTACCAGACAACCA	
gfp QRT-PCR R	GGTCTCTCTTTTCGTTGGGATCT	
p28 RT-PCR F	AATGCACAGGTACAGGAGAAACTAAAT	
p28 RT-PCR R	CACACTCATAATGTTTTCCAGTCAATT	
ama1 RT-PCR F	TATGGGTCCAAGATATTGTAGTAATAA	
ama1 RT-PCR R	GAATTAGCTTTACCATAAATATCTGC	
chit RT-PCR F	GCCCGCCCAGATGTAATTATA	
chit RT-PCR R	TGCCAAATTCCTACACCATCG	
ctrp RT-PCR F	AGAGAAGAAGATTGCCCAACAG	
ctrp RT-PCR R	ATCGGATCATTTGCATCGATAAC	
soap RT-PCR F	TCGAAGGAGCAAGGAAAAATTCC	
soap RT-PCR R	ATGAACAGCTACATTCTTCGGTC	
warp RT-PCR F	ATGAAGAGTGTTAAAGGAATAACATATAT	
warp RT-PCR R	CAGACGTCAGTGAAGAAGGATT	
maebl RT-PCR F	GAATTGAAGAAGCTAAGAAAGCAG	
maebl RT-PCR R	TTCTTCGTACCATCTTCCTCC	
misfit A F	*TTGGGCCC*AAGATGATAGTAATTATAAAGAATTAGAAG	Disruption upstream target (A) *ApaI*
misfit A R	*CCAAGCTT*GAACAATAATACTGTTACTAGTGG	Disruption upstream target (A) *HindIII*
misfit B F	*TGAATTC*TTGTTGCATATTTAGCAGTGAATTAG	Disruption downstream target (B) *EcoRI*
misfit B R	*GGGGATCC*GAATCAAATTCTGCTTCATCCATG	Disruption downstream target (B) *BamHI*
misfit A INT F	CATAAATTTTTACGATGAGGTA	38 bp upstream of *misfit A*
misfit WT R	GCCTGATCAACATATGAATCATTG	332 bp downstream of *misfit A*
tgdhfr-ts 5'UTR R	GATGTGTTATGTGATTAATTCATACAC	200 bp into the 5'UTR of *tgdhfr-ts*
misfit-myc F	*GGGGTACC*CAGAAGGAAGATTAAGTTGTATGG	Tagging 3' target *pbmisfit ApaI*
misfit-myc R	*TTGGGCCC*AGCAGAAGAATCAAATTCTGC	Tagging 3' target *pbmisfit KpnI*
Misfit-myc Seq F(1)	CAACAGTTCCACTTTCAAAAACTG	Sequencing (Tagging construct)
misfit-myc Seq F(2)	TGGAAACAAACCTAATTACGAAGTT	Sequencing (Tagging construct)

Where appropriate, target restriction sites are shown as underlined italics and restriction site overhangs (to ensure efficient cutting) are in italics. The appropriate restriction enzyme is presented in the description column. F, forward; R, reverse. All primers are listed in a 5′ to 3′ direction.
