# Supplementary figures and images for: Paternal Effect of the Nuclear Formin-like Protein MISFIT on Plasmodium Development in the Mosquito Vector
Source: PLoS Pathog. 2009 Aug 7;5(8):e1000539. doi: 10.1371/journal.ppat.1000539 (PMC2715856; doi:10.1371/journal.ppat.1000539)

**A**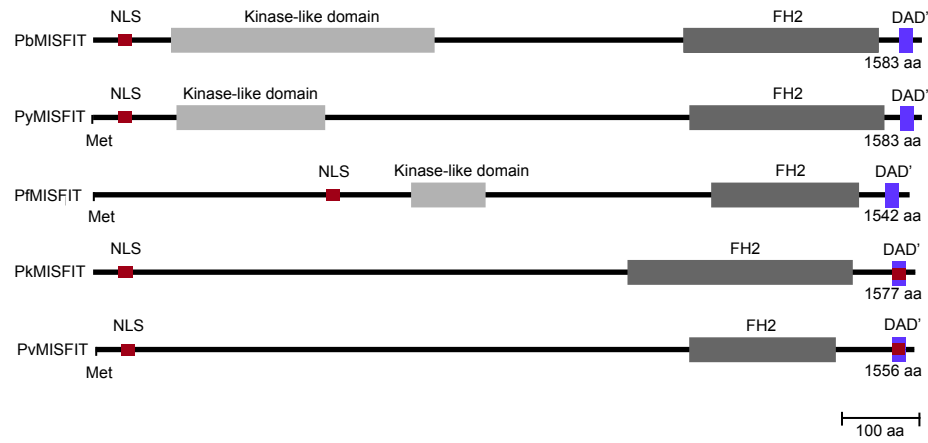**B**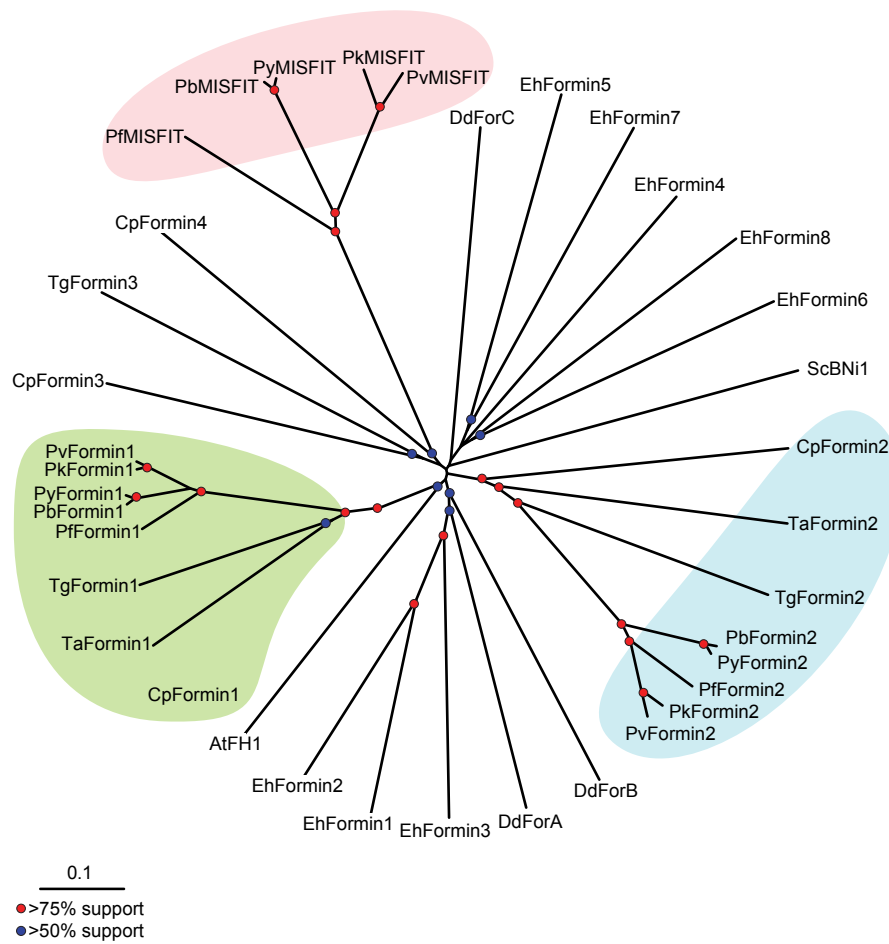

Supplement: Figure S1 — MISFIT structural features and phylogenetic analysis of the FH2 domain of apicomplexan formin-like proteins. (A) Schematic representation of protein features of PbMISFIT (Pb000064.01.0) and its P. yoelii (PyMISFIT; PY00811), P. falciparum (PfMISFIT; PF14_0035), P. knowlesi (PkMISFIT; PKH_134310, refined annotation) and P. vivax (PvMISFIT; Pv086245) orthologues. NLS, nuclear localization signal (red box); FH2, formin homology 2 domain (dark grey box); A putative kinase-like domain (light grey box) with unclassified specificity is predicted for P. berghei, P. yoelli and P. falciparum MISFITs. NLS was predicted by NucPred (http://www.sbc.su.se/~maccallr/nucpred/) and PredictNLS (http://www.rostlab.org/services/predictNLS/). The C-terminal region of MISFITs (DAD′) resembling the basic region of the Diaphanous-autoregulatory domain (DAD) of DRFs proteins is indicated. NLS sequences are predicted at the same region in P. knowlesi and P. vivax. A MISFIT orthologue also exists in Plasmodium chaubaudi, which due to poor sequence quality is not included in this analysis. (B) Phylogenetic analysis of the FH2 domains of MISFIT, Plasmodium Formin1 and Formin2 and other apicomplexan formin-like proteins. Red and blue circles show 75% and 50% bootstrap support for groups, respectively. PfFormin1 (PFE1545c), PfFormin2 (PFL092w), PvFormin1 (PV079720), PvFormin2 (PV123615), PyFormin1 (PY01292), PyFormin2 (PY01855), TgFormin1 (20.m05986), TgFormin2 (20.m03963), TgFormin3 (31.m00924), TaFormin1 (TA03495), TaFormin2 (TA09030), CpFormin1 (cgd6_4150), CpFormin2 (cgd8_2450), CpFormin3 (cgd8_1500), CpFormin4 (cgd2_3850), Bni1p (NP_014128), AFH1 (NP_189177), DdForA (AB082542), DdForB (ANB082543), DdForC (AB082544), EhFormin1 (XP_653752), EhFormin2 (XP_656030), EhFormin3 (XP_653884), EhFormin4 (XP_651696), EhFormin5 (XP_650406), EhFormin6 (XP_652294), EhFormin7 (XP_653981) and EhFormin8 (XP_650130). Tg, T. gondii; Ta, Theileria annulata; Cp, C. parvum; Sc, Saccharomyces cerevisiae; At, Arabid [file ppat.1000539.s008.pdf]

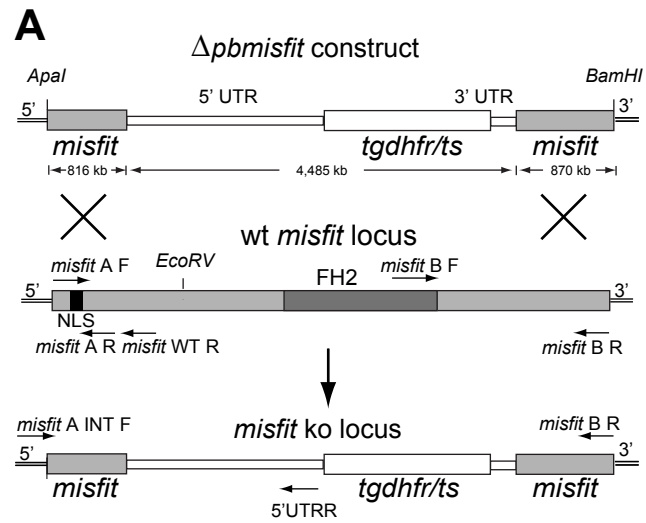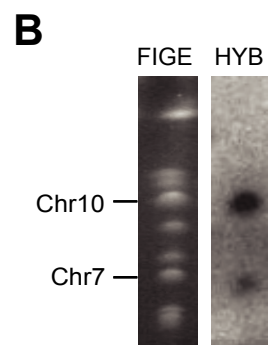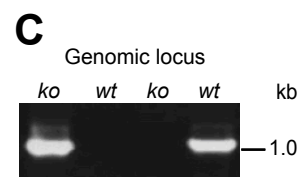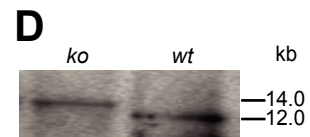

Supplement: Figure S3 — Generation of transgenic Δpbmisfit parasite. (A) Schematic representation of the pBS-tgdhfr/ts pbmisfit disruption vector, and the native and disrupted pbmisfit locus. The disruption vector carries pbmisfit targeting sequences, which flanks a tgdhfr/ts pyrimethamine-selection cassette. Integration of the ApaI/BamHI linerised vector results in the disrupted pbmisfit locus. pbmisfit coding region (open), tgdhfr/ts cassette (grey), predicted transmembrane domains (black). (B) Genotyping of Δpbmisfit. The integration of the disruption vector into the pbmisfit locus, held on P. berghei c507 chromosome 10, was confirmed by pulse-field gel electrophoresis where the blot was probed with tgdhfr/ts fragment. The signal at chromosome 7 derives from cross-hybridization with the native pbdhfr/ts locus. (C) PCR based analysis of the genomic DNA from wt and dilution cloned Δpbmisfit parasites show that the Δpbmisfit locus (misfit INT F and tgdhfr/ts 5′UTR) is only present in the ko line while the pbmisfit native locus (misfit INT and misfit WT R) is only present in the Pbc507 wt line. (D) Southern blot analysis in which the misfit PCR-amplified fragment (misfit A F and R) was used as a probe show that the insertion of the 5 kb tgdhfr/ts cassette and simultaneous deletion of 3 kb flanking sequence, resulted in an increase of 2 kb in fragment size of ko compared to the wt locus. (0.65 MB PDF) [file ppat.1000539.s010.pdf]

**A**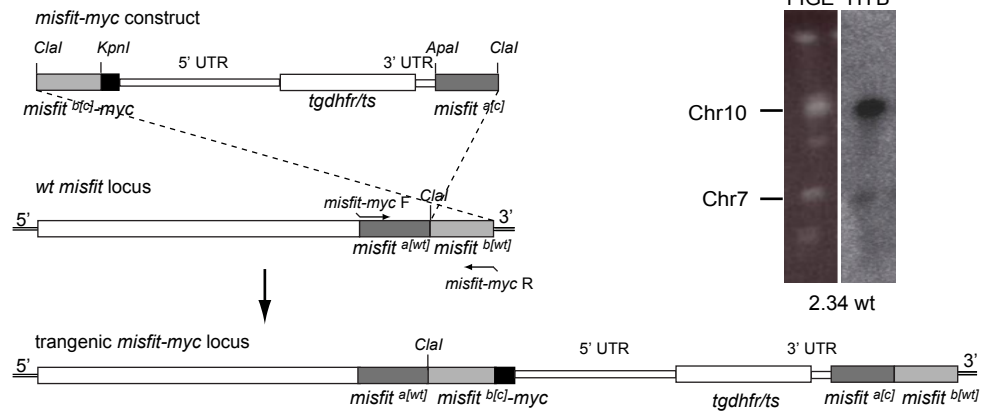**B**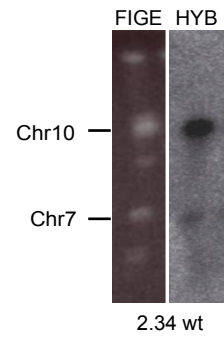

Supplement: Figure S4 — Construction of pbmisfit-myc transgenic parasite. (A) Schematic representation of the pBS-tgdhfr/ts pbmisfit-myc-tagging vector, the native pbmisfit locus and the resulting transgenic pbmisfit-myc locus. The tagging vector carries a C-terminal fragment of pbmisfit, with a unique ClaI restriction site in its centre, cloned in-frame with a double c-myc tag (black) and in tandem with a tgdhfr/ts pyrimethamine-selection cassette. Following construct linearization with ClaI, the pbmisfit-myc sequence is separated into two fragments, misfit a[c] and misfit b[c]-myc, where superscript [c] stands for cassette. Transfection of the linearized cassette on P. berghei (ANKA 2.34 strain) results in single homologous recombination and replacement of the last 942 bp of the native pbmisfit locus (misfit b[wt]) with its myc-tagged version (misfit b[c]-myc). (B) Genotyping of pbmisfit-myc. The integration of the tagging vector into the native pbmisfit locus in chromosome 10 was confirmed by pulse-field gel electrophoresis using the tgdhfr/ts fragment as probe. The signal at chromosome 7 derives from cross-hybridization with the native pbdhfr/ts locus. (0.59 MB PDF) [file ppat.1000539.s011.pdf]

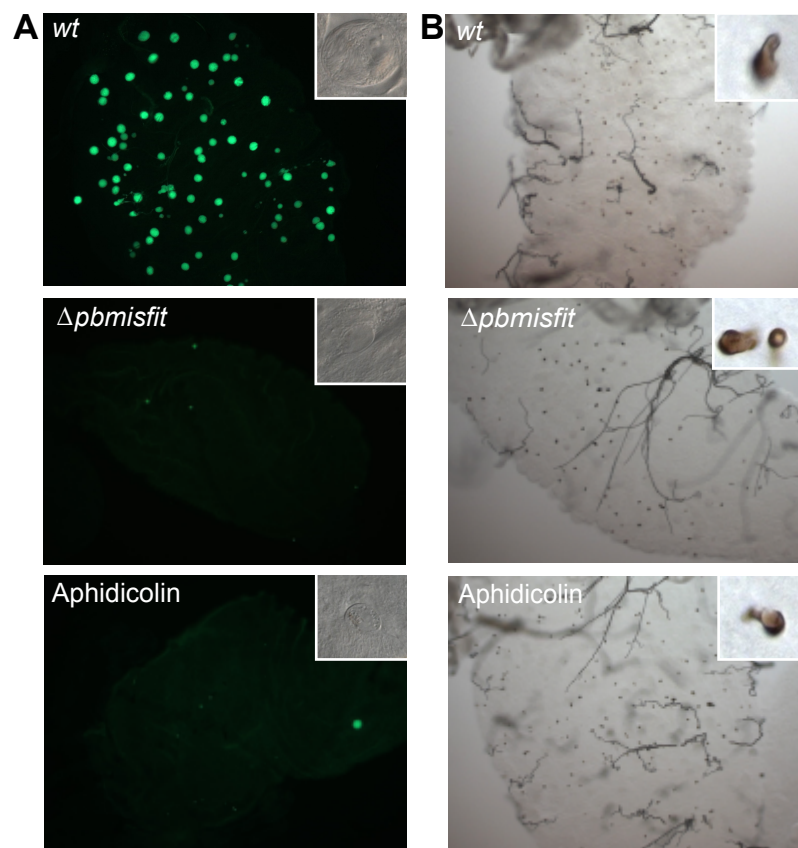

Supplement: Figure S5 — Phenotypic analysis and invasion assays of Aphidicolin treated ookinetes. (A) Fluorescent microscopy images of 7-day old GFP-expressing oocysts of wt, Δpbmisfit and wt Aphidicolin-treated parasites in A. gambiae midguts. (B) Microscopy images of wt, Δpbmisfit and Aphidicolin-treated ookinetes that are melanized immediately after invasion of CTL4 kd A. gambiae midguts. Aphidicolin treated ookinetes, like Δpbmisfit, do invade the midgut but fail to produce sporulating oocysts. Insets show oocysts and melanized parasites in high magnification. (3.92 MB PDF) [file ppat.1000539.s012.pdf]
