# Supplementary material for: Paternal Effect of the Nuclear Formin-like Protein MISFIT on Plasmodium Development in the Mosquito Vector
Source: PLoS Pathog. 2009 Aug 7;5(8):e1000539. doi: 10.1371/journal.ppat.1000539 (PMC2715856; doi:10.1371/journal.ppat.1000539)
Supplement: Figure S2 — Multiple sequence alignment of Plasmodium MISFIT proteins. Colored blocks indicate amino acid residues conserved among all species. The FH2 domain, a central conserved region and the DAD-like sequence (DAD′) are indicated. Alignment was performed by ClustalW and visualized by BioEdit Sequence Alignment Editor. (0.15 MB PDF) [file ppat.1000539.s009.pdf]

```
                    10        20        30        40        50        60        70        80
            ....|....|....|....|....|....|....|....|....|....|....|....|....|....|....|....|
PbMISFIT    -----------EKLKKLTGKLLYLLNKYRHAYLMSIDLINFYDEVIEKKD-DSNYKELEEFYKKKRPWEG-IIDEEMIIR
PyMISFIT    MAVLITLEKENEKLKKLSEKLLYLLNKYRHAYLMSIDLINFYDEVIEKKN-DSNYKELEECYKKKRPWEG-IIDEEMIIR
PkMISFIT    ---------ENVKLKRINEKLLSLLNRYRCAYLISLDLVNFYEEVIRRRCKENGGKDLLEGLKRRKQLND-TWEEEMVMR
PvMISFIT    -----MLDQENVKLKRINEKLLSMLNKYRCAYLMSLDIVAFYEEVIRRRCREGGGKDLLEGQKRRKQLND-TWEEEVVLR
PfMISFIT    MAPDTLLNKYISKFQKINDKLLGLVNKYRYAYFKALEVINHYEELVKRNGELFDDKELIEIYKKRRQCEECILEEEKEIR
Consensus               *::::. *** ::*:** **: ::::: .*:*::.:.    . *:* *  *:::  :    :**  :*

                    90       100       110       120       130       140       150       160
            ....|....|....|....|....|....|....|....|....|....|....|....|....|....|....|....|
PbMISFIT    KKLKNNLCYYEGKEHTKKIKTIETNDSEMLHSKGNDGNDSSNCS------------------------------LSGIKI
PyMISFIT    KKLKNNLCYYEGKEHTKKIKIIETGDSEILHSKG---NDSSNCS------------------------------LSGIKI
PkMISFIT    KKLKMKLPMNEGSIEVTQCGGIGQG-VDSAGRVSPPLVGKENCSEERYSSGD--------------------IPVDTTNI
PvMISFIT    KKLKMKLPGKEASFEVTLCGGVGEG-VDSARGVCPPLAGGEDCSDERDSSGDTPAEAAVETAVDTPTETPVNTPVDTLNA
PfMISFIT    KKLDREKDNNIMSTNYTKDYNINIERDNLKEENVHVVMND-----------------------------------ESVEQ
Consensus   ***. :      .  . .    :     :          .                                    .  :

                   170       180       190       200       210       220       230       240
            ....|....|....|....|....|....|....|....|....|....|....|....|....|....|....|....|
PbMISFIT    FKGDDSKLQDNNMENEDEK---------YNKNTSDYVSLGALDFSAYKEDSDGHIKYYEKNKTDIKVDKTKKNNN-----
PyMISFIT    FKGDDSKLQDNNMESEDGK---------DNKNTSDYVSLGALDFSAYKEDADGHIKYCEKNKTDIKVDKTKKNNN-----
PkMISFIT    VPTSVPKAPETIHHQGNQSSHAKSSAIIHESNKSNYVNLGALDFSAYKEDSEGCVKYFEKKKTQLSNDKGNTESC-----
PvMISFIT    VPTSATNAPHAAHQPGQGS----SNAILHERNKSNYVNLGALDFSAYREDSEGYVKYFEKKKTQLSNDKVSTEAEPN---
PfMISFIT    IKDNNPMIDHNTSCIVDIN----------EKNKSNYVDLGTLDFSAYKEDSQGCIQYYENKEDVQEQKQGKSQEVESKRH
Consensus   .  . .   .      : .          : *.*:**.**:******:**::* ::* *:::   . .: ..:

                   250       260       270       280       290       300       310       320
            ....|....|....|....|....|....|....|....|....|....|....|....|....|....|....|....|
PbMISFIT    ---NFSKYMHQSDVKKKNVLNQSIY-MKNNN------------VKSSQIRILPHIHKNIYNADNSFSKLNSAKKLNALNT
PyMISFIT    ---NFSKYMQQSDVKKKNVLNQSIY-MKNNN------------VKSSQIRILPHMHKSIYNPDNSFSKLNSSKKMNVLNS
PkMISFIT    -YDNSGRQLHQSEFRRRPLFNQSVYGSKQSV------------IKGALNRELPFSHKSIYMDK--HSKLQGAKKEDILTN
PvMISFIT    -YNNAGRQLHQSEFRKRPLFNQSVYGSKQSV------------IKASPSRVLPYSHKSIYMDR--HPKFQGAKKEDALTN
PfMISFIT    MRVNSSRYESQYGIKNRNILKQSIYVKQNNNSNNMKSVSGSRYVPNKSVINIDKSFKVTHNRNYENLSKKSIYNNNNINN
Consensus      * .:   *  .:.: :::**:*  ::.             :       :   .*  :       . :.  : : :..

                   330       340       350       360       370       380       390       400
            ....|....|....|....|....|....|....|....|....|....|....|....|....|....|....|....|
PbMISFIT    YKSVYIN-----------SNIMHKN-IGMDKKIVKNELNKNEFLQNNN--LKGLNKSVYIGRRE-KSTCATSNSIIVPNR
PyMISFIT    YKSVYIN-----------SNIMHKN-IGMDKKIVKNELNKNEFLQNNN--LKGLNKSVYIGRRE-KSMCATSNSIIVPNR
PkMISFIT    -RSVYVTSGRGIGSGIGMNNLLSKN-MGLARKVVKNELHKGGSIAPSHRPITNLNKSVYVETKG-AHNRDSHLSEGVG-C
PvMISFIT    -RSVYVS---GSGSGTGMSNLLSKH-VGVPRKVVKNEPHKGSFIAPSHRPITHLNKSVYVDTKG-AHNRGSNLSEGLG-G
PfMISFIT    NNNINNNNNNNNSNNNNLVNKIGVGGINKKGGEKKMDVHKSTYIGSNNKLLNNMNRSIYIGAKKNINSNNININKNSNIH
Consensus    ..:  .            * :    :.      * : :*.  :  .:  :. :*:*:*:  :          .

                   410       420       430       440       450       460       470       480
            ....|....|....|....|....|....|....|....|....|....|....|....|....|....|....|....|
PbMISFIT    NNRMSGPINESLILTSKNSLINKDGIKKQELLNKKMSVIKANNTHNNNIPLKKMNTLSILKNNNYISNKNGLNKSIYFSR
PyMISFIT    NNRMSGPINESLIHTNKNFLINKDGIKKQELLNKKMSVIKANNTHNNNIPLKKMNTLSTLKNNNYISNKNGLNKSIYFSR
PkMISFIT    SNKASLLTRDNLKKGESAVNKKVGMLNRPSGFQNSVYVKRMNSTIGGSSGVVGGGPISFSPPD---ARKGGPNKSVYMR-
PvMISFIT    TNKATLLSRDNLKK--------VSTVSRPSGLQNSVYVKRMNSTIG--SGGGGGGPISFSPPH---ASKGAPNKSVYMR-
PfMISFIT    NNIHNFKNPYDNNTSLLSTHMNTVVINKEKDMNDTGNNKNVTNAYTQNSLYIKKGKSFNVSNNSLYINKNICQSSVYNK-
Consensus   .*  .     .              :.: . ::..    . ..:          .       .     *.  :.*:*

                   490       500       510       520       530       540       550       560
            ....|....|....|....|....|....|....|....|....|....|....|....|....|....|....|....|
PbMISFIT    SNTLMSNDKKKKTNDSYVDQAKVGTVGDSNLNRKAEHINNLNFWGKNEVLDVEISKEIGIAIKDVETENTEQNSKKIFID
PyMISFIT    SNTLMSNDKKKTSNDTYIDQAKFGTVGDSNLNRKTEHINNLNFLGKNEVRNVEISKEIEITIKDVETENIEQNIKKTFID
PkMISFIT    SNTFAISNKKRKTSEGGENFKCSSANDTVKAEGKVTHLYDGVAVQREKMSTVGLCIEEENNLDENIKENK----------
PvMISFIT    SNTLAIGNKKRKTSEGEESFKCLSANEKVKAEGKVTHLHDGMAAEGEKTSSGDGCIEEENTLDGTLEEEK----------
PfMISFIT    SNTIIYNAKKRKTNEKEKEEDKDGYKNNEIVCTNKNKESNEDNNSDERNKNKHLTNNIINKENNKIYIKN----------
Consensus   ***:  . **:.:.:   .    .         :  :  :      :.        :     .     :

                   570       580       590       600       610       620       630       640
            ....|....|....|....|....|....|....|....|....|....|....|....|....|....|....|....|
PbMISFIT    EKLGIEKSDKINFPNVQKLSYSPLTLEDIEKLIKDEEKDKEISVNNNLNQSLIKENDFACMKKNICIDKDNLKNLVCLPE
PyMISFIT    EKLGIEKSDKINFPNVQKLSYSPLTLEDIEKLIEDEEKDTGISVNNNLNPSLTKENDGICMKKNICIDKDDLKNLVCLPE
PkMISFIT    ------RKESLDMVVYFHHSYEPLTLENVEFLMGNEIIDGEHARGGNSLGDNQEGDVGRDDRVHISSVENGKRGEYYMKE
PvMISFIT    ------RKESPNMVAYFHLSYEPLTLKNVELLMGSEIIDGEHSLGGSSLGDDT------------SSVENAKRGKDYTRG
PfMISFIT    -----DISKNISYIYDNLNTYIPITLEHIKNLIEWDDADYEDIKKYTFGNILHLKDTNYNILEGIDLECDKTKCMEKICE
Consensus          ... .       :* *:**:.:: *:  :  *       .                      :  :        &
```

```
                    650       660       670       680       690       700       710       720
             ....|....|....|....|....|....|....|....|....|....|....|....|....|....|....|....|
PbMISFIT     KNQTYI------KIEKNCDDNGENISQINELKKEDKINGDNNKNTNFGASTINSENINNEVVGYIENPLSDNLIKVEKKI
PyMISFIT     K--------------KNCDDNGENISQVNGIKKENKINEDNNENSNLLANTINSEIINNEVVGHIGKPLSDNLIKVEKNI
PkMISFIT     CLAR-----------TEGYSP------EAEEHNAGFVSFKKETLSKLVQKGEEKSSIHLGEITPVGDDMDGHSNEKSAHS
PvMISFIT     WPPR-----------TEGYSAGGEEPQEGEKRNAGDVPLKKEAVSNLAQKGEEKSSLHLGEVTPVGGDMDSHS-------
PfMISFIT     KTNEDVPTYKNMYPIYKGDNMKGELYIKDEKCNIQNINNKSDDKKKCYLTKYKNVHIYKSDISSCPNEYKNNVDESNEQQ
Consensus                    :  .            :   :  ..:  .:   .  :.  :    :       ..:

                    730       740       750       760       770       780       790       800
             ....|....|....|....|....|....|....|....|....|....|....|....|....|....|....|....|
PbMISFIT     EEKSS---EYALVKKEGNISMENNSNKMSYNEKCKITKDLEEENMDIDNKKDIKNIENINTKDN----------------
PyMISFI      EEKSS---EYALIKKEENISMENNSNKMSYNEKCKITKEVEEENMGIDNKKDIKNIENINTKDN----------------
PkMISFIT     GEENFRMNHNSLLGREGTISSGERAKEMDKDHPEGG-RKMKEDKPDMERKNKKEPMKPLSHHMGSEEMGRLNNDSCGEEE
PvMISFIT     GGEKLGIKRASLLGREGSIPPGDRVNGLDKDHPVGG-SNMKEDDPDMERRNKNDHIQPLNHLSG----------------
PfMISFIT     EDSFVRERDNANLDGSNQVHIKKINDNNSNMSTEYTCEQISLKNNKNDGERNHVNKLIIDKVPIN---------------
Consensus      .       : :  .  :   .  .  .         .:. ..   : ...      :.

                    810       820       830       840       850       860       870       880
             ....|....|....|....|....|....|....|....|....|....|....|....|....|....|....|....|
PbMISFIT     -------------------VKKLDYSAISCGHEEINRKNIILNINDEIKEKIMERECVSGKVGNNSEENVFKKMSEKK--
PyMISFIT     -------------------VKKIDYSVISCGHEEINRKNIILNINDEIKEKIMEREGVSGKIENNSEENVCKKMNEKK--
PkMISFIT     GEPRVKGGEGIGGIFTVVESGQLGRTKQTRSEMPLKFQSIILSINDEIKRKLKGG-IEKGEIMNASKGAESNTSGNIS--
PvMISFIT     -------------------CDQIGRAKRTSSEMPLKFQNIILSINDEIKRKLQGGCSEKGEVSSASKCKEANTSGAKP--
PfMISFIT     -----------------------KLIKEPSDDISLKYKNIIFNINDEIKKKVNKEKNEEINEHVESLSKVCNKPRNIKNV
Consensus                                    . ..  :: :.**:.******.*:      . :    *     :.

                    890       900       910       920       930       940       950       960
             ....|....|....|....|....|....|....|....|....|....|....|....|....|....|....|....|
PbMISFIT     MLISENIRKNLMSSLEKETG---TKCSNINNRANLNIEIDLDCEYVSLDIVNIDLAINEGLYNYKKNEESYIECDEQIKD
PyMISFIT     MLISENIRKNLMSSLEKETG---TKCSNVNNRANLNIEIDPDFEYVSLDIVNIDLAINEGLYNYKKNEESYIECDEQIKD
PkMISFIT     DFTKSLKRSMLLCEGEKKNEGVEIERNINNNRANLHIEISTSVGYISLDMIDLDKAMYEGLFSCKDMEGSLIPSEENVKD
PvMISFIT     SFTASVRKNLLPC-GENTNAGVEIERSVNNNRANLHIQMSTTVGYVSLDMVDLDKAMYEGLFLFKDVERSFIPSEQNVKD
PfMISFIT     PFPSSLKKNILLESQEKKKN-------PINNRCPLHLDINVDVKYITLNTIDIDETINKGVCPYKDMEDNYISSEEHIKD
Consensus     :  .  :. *    *: .          ***. *::::.    *::*: :::* :: :*:   *. * . * .::::**

                                                                Conserved region
                    970       980       990      1000      1010      1020      1030      1040
             ....|....|....|....|....|....|....|....|....|....|....|....|....|....|....|....|
PbMISFIT     IKIYNDILKSEKE----ICSDLLFIKKENQFN------NNSVILFKSSNEIKNEISKYVGMSIFDCIN-KFIIPTNSLVN
PyMISFIT     IKIYNDILKSEKE----ICSDLSFIKKENQFS------NNSVILFKSSNEIKNEISKYVGMSIFDCIN-KFIIPTSSLVN
PkMISFIT     IAMYDEILKEEAK----GNFNNISPYSINNENKLMFSRRSQISLFKSSQEIKNEMMEFLGVSIFYSINMESLFPSVSLVN
PvMISFIT     IAMYDEILKGEAK----GSSNNTSPHSKINSNQLGLSGTSQRSLFKSSQEVKNEMMGVLGVSIFYSVNVESLFPSVSLVN
PfMISFIT     IIIYSDILKTEKETFLYNPNDIEKYDNNDDSNNNVRKIPDTIIHFKSSNEIKNDIIKLVGISIFDCIH-KSYEPTKSLIN
Consensus    * :*.:*** * :       :     .  : .       .    ****:*:**::   :*:*** .:: :   *: **:*

                                  Conserved region
                   1050      1060      1070      1080      1090      1100      1110      1120
             ....|....|....|....|....|....|....|....|....|....|....|....|....|....|....|....|
PbMISFIT     DNRLSIWFTKKKKNNN----NMNRSKDNNFDRIENYNDDSKIRRNFIFKKKSEGRLSCMGYFSQPITIMLSSLKRRSEFG
PyMISFIT     DNRLSIWFTKKKKKNN----NMNRSKDNNFDRIENYNDNSKIRRNFVFKKKSEGRLSCMGYFSQPITIMLSSLKRRSEFG
PkMISFIT     DEKLALWFTKRVREKN----MNKNVLAKSYYRNNNP-----------LGGRIKKRLSCMGYFTQPVTIMLCSLKRQSEFK
PvMISFIT     DEKLSLWFTKRVREKN----AHKHALAKSFCRGSNP-----------FGGRIKKRLSCMGYFTQPVTIMLCSLKRQSEFK
PfMISFIT     DENLEIWFTKREKNKNDDMINNMRGNIVQLNYGQDKELVFEKKKKKKKKNIINKRISSMGYFSQAVVIMLCSLKRQNEYK
Consensus    *:.* :****: :::*       .    .    .:                 : *:*.****:*.:.***.****:.*:

                                Conserved region
                   1130      1140      1150      1160      1170      1180      1190      1200
             ....|....|....|....|....|....|....|....|....|....|....|....|....|....|....|....|
PbMISFIT     NLKNIITSIILCTCDSSMLEIILHTIPDENSKNYQLWKTSIKKLYTLIG-DRKKDILRNLIFSD----------------
PyMISFIT     NLKNIITSIILCTCDSSMLEIILHTIPEENSKNYQLWKTSIKKLYTLIG-DRKKEILRKMIFSD----------------
PKMISFIT     NLKKIISAVVLCTCDSTTLEKMLHTIPEASSKNFSLWTEALHRLESQLGLKRKRRIIRKLLRAGGGEDTRRDDERKGMGL
PvMISFIT     NLKKIVSAVVLCTCDSTTLEKILHTIPEASSKHFSLWTEALHRLESQLGPKRKRSIIRKLLRAGGGEDARKGDNRENAHL
PfMISFIT     SLKKIIASIILCTCDSSCLEKFLHTIPDENSKNYTLWKETLNKLTSYFGPNKCDEIVERLMIIE----------------
Consensus    .**:*:::::******: ** :*****: .**:: **. ::::* : :* .:   *:..::

                   1210      1220      1230      1240      1250      1260      1270      1280
             ....|....|....|....|....|....|....|....|....|....|....|....|....|....|....|....|
PbMISFIT     ----KDKTCETHEKDEREGYDENNLIEDKNKYNELNLKTYDFMNTMDNNGYRNFFNSSFSSEAGGNTPINQNNYFSTVPL
PyMISFIT     ----KDKTFETDEKDEGEGYDENNLIEEKNKYNELNLKTYDFMNTMDNNGYRNFFNSSFSSEAGGNTPINQNNYFSTVPL
PkMISFIT     ANPRDSDPYVEESGSDENGEDDDQTGRKKKQDDQFELKTYDFMKGMN--TYDGCLSDSFSSKG---VEELDNATHTSMSS
PvMISFIT     ANPRDSDPYGEDSESDDNDEDDDQTGRKNKHDDQFELKTYDFMKGMN--TYDGCLSDSFSSKG---VEQSDSATHTPVSS
PfMISFIT     -------------EDEYMNKSKKECREKNDYYEGDNIKSNKNIYNIYNNNDEKDIIKHCKLDN-DMEFFDDHMNNLSFPF
Consensus                  .:  . ...:  ..:.  :  ::*: . :  :        : .  . .        :     ...
```

```
                                                         FH2-domain
                  1290      1300      1310      1320      1330      1340      1350      1360
           ....|....|....|....|....|....|....|....|....|....|....|....|....|....|....|....|
PbMISFIT   SKTDIKTEKYEEGKENNINILEDEKFCLFICTINDIHKRFQYLLLIENYDFIYSDLIKHIQNKLNNIDLIINKHMLLKQL
PyMISFIT   SKADVKTEKYEEGKENNINILEDEKFCLFICTINDIHKRFQYLLLIENYDFIYSDLIKHIQNKLNNIDLIINKHMLLKQL
PkMISFIT   SRGTLIQEKQ---NEENFETYEDEEFCLFICTIPNVHKRFRYLVLIENFHFIYEDLLKHIHNKLMIIELIASKHLLLKQL
PvMISFIT   SRGTLTREKQ---NEEHFETYEDEEFCLFICTIPNAHKRFRYLLLIENFNFIYEDLLKHIQNKLITIELIASKHLLLKQL
PfMISFIT   ECNEEHNKKYNDIQEEDDEIYEDEQFCLFICRIKNVHKRFGYLLLIDNFDFVYEDLLKNIRNKLSSIELILTKHILLKQL
Consensus  .      :*    :*:. :  ***:****** * : **** **:**:*:.*:*.**:*:*:***  *:** .**:*****

                                          FH2-domain
                  1370      1380      1390      1400      1410      1420      1430      1440
           ....|....|....|....|....|....|....|....|....|....|....|....|....|....|....|....|
PbMISFIT   FCNVLFLCNWLNEPKCFKWFQWNDVFNKLYNLNGFLENGKISRERCILLLLAEHTGEIFTEKELNELKKTSKLHIKDLYD
PyMISFIT   FCNVLFLCNWLNEPRCFKWFQWNDVFNKLYNLNGFLENGRISRERCILLLLAEHTGEIFTEKELNELKKTSKLHIRDLYD
PkMISFIT   FCNILFLCNWLNEPKRYRWFQWDTVVKKVEMLHGYLENGRISRDRCMLVLLAEHTGEIFSDKELQELKKVSKFHLKDLYD
PvMISFIT   FCNILFLCNWLNEPKTYRWFQWDTVVRKVEMLHGYLENGRISRDRCMLLLLAQHTGEIFSDKELHEMKKVSKFHVKDLYD
PfMISFIT   FCNILYICNWLNKPRKYEWFEWNMVVKKLRKLYGYSENGRISKERCIMLILAKHTGEIFTNKELYFLKKISTFYIKDLYD
Consensus  ***:*::*****:*: :.**:*: *..*:   * *: ***:**::**::::**:******::***   :** *.::::****

                                          FH2-domain
                  1450      1460      1470      1480      1490      1500      1510      1520
           ....|....|....|....|....|....|....|....|....|....|....|....|....|....|....|....|
PbMISFIT   KSIDFINCFLELKNQLETEEFKKSCCIFSS-ELEDVGVEKCSDNFLNDKVLKNDKFLEKVKDFVKKYYKQMVYIIWNLVL
PyMISFI    KSIDFINCFLELKNQLETEEFKKSCCIFSS-ELEDVEVEKCSDNFLNDKVLKSDKFLEKVKDFVKKYYKQMVYIIWNLVL
PkMISFIT   RSIDFINSFLELRGEMGTSEFARSCCVGETTGEVNAEVDMETDEE----NLLKDKFLEKVHEFVEKVYGKMLLIISRLVL
PvMISFIT   RSIDFINSFLELRGEMGTAEFARSCCVGEVPAEVEAEVEAEAEEEDAVGNLLKDKFPEKVHQFVEEVYGKMLLIISRLVL
PfMISFIT   KCIDFINTYLEIKNDIDTEEFLDSCCIHNLKNEIKDNNTSYNHSS---KYFLHDKFLGIVKKFVQKNYPKILYIIWNIVL
Consensus  :.***** :**::.:: * **   ***: .      .      ..      :  ***    *:.**:: * ::: ** .:**

                     FH2-domain
                  1530      1540      1550      1560      1570      1580      1590      1600
           ....|....|....|....|....|....|....|....|....|....|....|....|....|....|....|....|
PbMISFIT   LIKKYLIVMIWLGDVKPFYPLFSYLDE-GKKIKYSEDLFVNLCHFFESYNKYFNIVKQSQEELQKGIGDTSYQGQNEKTK
PyMISFIT   LIKKYLIVMIWLGDVKPFYPLFSYLDE-GKKIKYSEDLFVNLSHFFESYNKYFNIVKKSQEELQKGIGDTSDQGQNEKTK
PkMISFIT   LIKQYLALIIWLGDIRPFYPLFSYVDE-TRKVKYSQDLFVNLVAFFESYNKYFNMIQKDNE-----------IGKNSERK
PvMISFIT   LIKQYLALVIWLGDIRPFYPLFSYVDK-TRKVKYSQDLFVNLVAFFESYNKYIHMIQKDNE-----------MGKNSERK
PfMISFIT   LIKQYLVIILWFNDIKPFFPLFNYPNDDNKQNKFLQDFFVNFVHFFENYNKYIDILKKENLN-----------EKIKNTS
Consensus  ***:** :::*:.*::**:***.* :.  :: *: :*:***:   ***.****:.::::.:            : .: .

                  1610      1620      1630      1640      1650      1660      1670      1680
           ....|....|....|....|....|....|....|....|....|....|....|....|....|....|....|....|
PbMISFIT   NIALNNSDDFIFSADYFEKNTQKEANGIANDSNYTNFKGGNKPNYEVCELRKNKIVEMNNRNYNNGSEYTANNSYIENND
PyMISFIT   NIALNNSDDFIFSADYFEKNIQKESHEIANDSNYTNFKDGNKPNYETCELRKNKIVEMNNRNYNNDSEYIENNSYIENND
PkMISFIT   RHALLDG----------------------GTSGCSFRPGFYSPTVDGASVR----VDRNLRGTNGLEHGNGGKKAVN---
PvMISFIT   RHALLDG----------------------DTSSCSSRPGFYSPTLDDASLQ----VGGNLRGTNGLERPGGARRAVDGNA
PfMISFIT   NDIFCDTSN-------------------------------------------INININIPSSENEHNTLTNN-------
Consensus  .  : :                                              :  *  . :.        ..

                                      DAD'
                  1690      1700      1710      1720      1730      1740      1750
           ....|....|....|....|....|....|....|....|....|....|....|....|....|....|...
PbMISFIT   ISIKNGNNVKGMLHFMKNNGNLKTENYELKHRKSLTNTIDYGCEIRRKSIEKEKGNNNKFIKGASFENMDEAEFDSSA
PyMISFIT   ISIKNCNNEKGMLDFMKNNDNLKTENYELKHRKSLTNTIDYGCEIRRKSIEKEKGNNNKFIKGASFENMLEAEFDSSA
PkMISFIT   -----------NMYGDSLFDSSKALNTEVKKRKSLTNTVDYACEVKRKSVEKERRRRS--VRQTSFEKSCEEEFHLSD
PvMISFIT   GGRKSDHGGAVNMYGDPFGDSSKALNTEVKKRKSLTNTVDYACEVKRKSVEKERRRRS--VRQTSFEKTCEADFELSD
PfMISFIT   ----------YDIYTNNRLSHQSTYNAETKKRKSLTNTIDYACLLKRKSAEHKETMEIS--KPSNAGNFSDVEFEPSD
Consensus             :       .   .: *  * *:*******:**.*  ::***  *::.   .    : :.   :   : :*. *
```
